# CLUMondo-BNU for simulating land system changes based on many-to-many demand–supply relationships with adaptive conversion orders

**DOI:** 10.1038/s41598-023-31001-3

**Published:** 2023-04-05

**Authors:** Peichao Gao, Yifan Gao, Xiaodan Zhang, Sijing Ye, Changqing Song

**Affiliations:** 1 State Key Laboratory of Earth Surface Processes and Resource Ecology, Beijing Normal University, Beijing, 100875 China; 2 Center for Geodata and Analysis, Faculty of Geographical Science, Beijing Normal University, Beijing, 100875 China

**Keywords:** Ecology, Ecological modelling, Ecosystem ecology, Environmental impact

## Abstract

Land resources are fundamentally important to human society, and their transition from one macroscopic state to another is a vital driving force of environment and climate change locally and globally. Thus, many efforts have been devoted to the simulations of land changes. Among all spatially explicit simulation models, CLUMondo is the only one that simulates land changes by incorporating the multifunctionality of a land system and allows the establishment of many-to-many demand–supply relationships. In this study, we first investigated the source code of CLUMondo, providing a complete, detailed mechanism of this model. We found that the featured function of CLUMondo—balancing demands and supplies in a many-to-many mode—relies on a parameter called conversion order. The setting of this parameter is a manual process and requires expert knowledge, which is not feasible for users without an understanding of the whole, detailed mechanism. Therefore, the second contribution of this study is the development of an automatic method for adaptively determining conversion orders. Comparative experiments demonstrated the validity and effectiveness of the proposed automated method. We revised the source code of CLUMondo to incorporate the proposed automated method, resulting in CLUMondo-BNU v1.0. This study facilitates the application of CLUMondo and helps to exploit its full potential.

## Introduction

The sustainable management and conservation of land resources have been central to human society^1,2^, as the resources are limited but provide the ultimate basis for “more than 95% of human food supplies, the greater part of clothing, and all needs for wood, both for fuel and construction”^3^. A critical focus of the management and conservation is on land-use and land-cover change, or land change for short e.g.,^4–6^. The land change represents the transition of land resources from one macroscopic state to another. More importantly, this transition is a crucial driving force of environmental and climate change locally and globally, which in turn affects land resources^7,8^. As a result, many efforts have been devoted to estimating future land changes in different scenarios and employing these estimates to inform management and conservation policies e.g.^9–11^.

Given the importance of future land change estimates, tools have been actively developed for their generation. These tools are called land change simulation models, classified into spatially aggregated and spatially explicit. Spatially aggregated models estimate future land changes in terms of quantity (i.e., composition). Such models usually serve as an essential component of integrated models for simulating coupled human and natural systems^12^. A typical example is the Global Change Assessment Model^13,14^, a maker model for the famous Shared Socioeconomic Pathways^15,16^. Its land use component produces future areas of more than 60 land types (e.g., rainfed cornland with high fertilizer or irrigated rice land with low fertilizer) at the spatial resolution of 235 water basins worldwide. Spatially explicit models generate the estimates of future land changes in configuration (if the composition information is an output of another model), or sometimes both configuration and composition. Examples of such models include cellular-automata-based models—e.g., Future Land Use Simulation (FLUS) model^17^ and Land Use Scenario Dynamics-urban (LUSD-urban) model^18^—and suitability-based models, e.g., Conversion of Land Use and its Effects at Small regional extent (CLUE-S) model^19^ and its latest version CLUMondo^20^. The output format of such models is usually a raster dataset, whose spatial resolution can be as fine as that of input data. Therefore, spatially explicit models are more specialized in land change simulation and are widely used.

**Figure 1:**
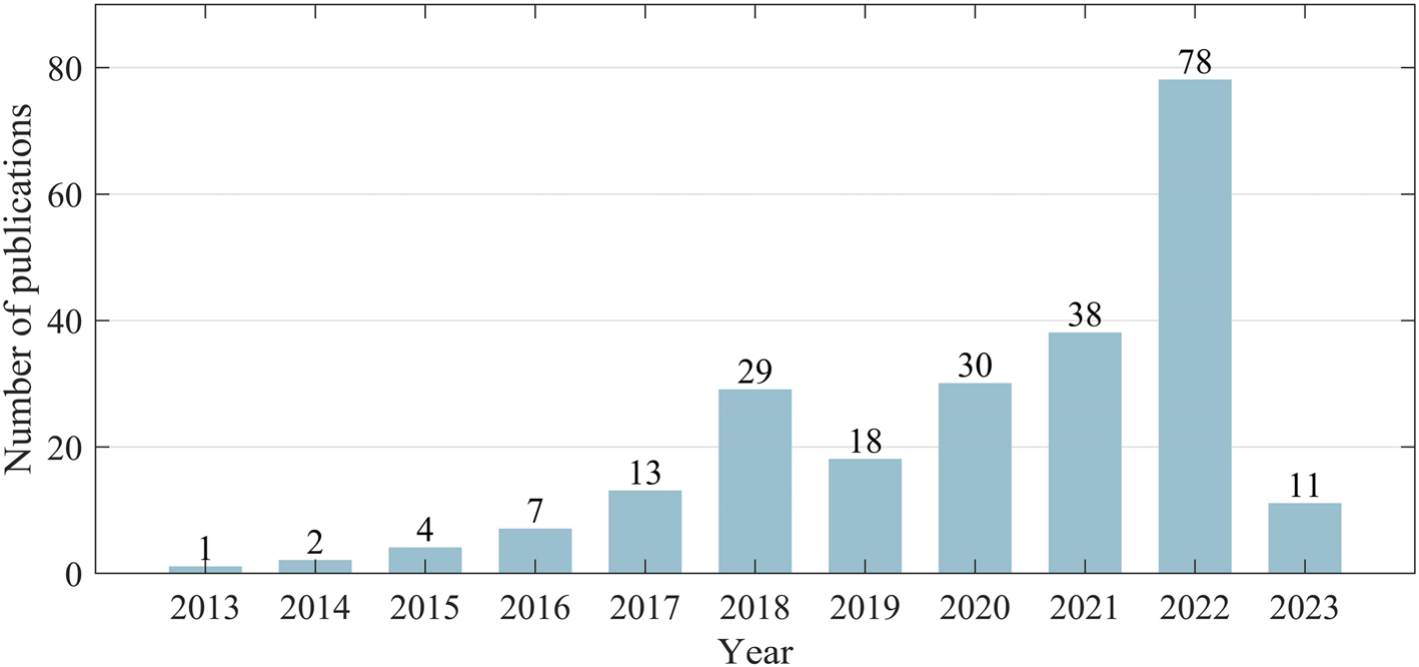
Increasing number of publications retrieved from Google Scholar with the keyword “CLUMondo” by 2 Feb 2023. When searching Google Scholar, we used double quotes to signify a phrase search and unchecked the “include citations” box.

Among all spatially explicit models, CLUMondo is the only one that simulates land change with many-to-many demand–supply relationships. Specifically, spatially explicit models balance a pre-defined, aggregated demand and the sum of corresponding, spatially explicit supply, although with different simulation strategies and techniques. Usually, the aggregated demand is specified as the areas of different land types e.g.^21–23^. In this case, the model adjusts the original types of land grid cells (hereafter cells), according to some mechanism, to supply the same areas of land types. The resultant relationship between the pre-defined demand and the corresponding supply is one-to-one; in other words, the demand for the area of a specific land type can only be met by supplying that type (i.e., by allocating that type of cells). Sometimes, the demand also involves the amount of goods or services, e.g., population, food production, or ecological/economic benefits. In practical simulations, however, such non-area demands are transformed into the area demands for different land types to achieve one-to-one demand–supply balances e.g.^24,25^. The only exception is CLUMondo, where the balance can be achieved in terms of not only land type areas but also the amount of goods or services e.g.^26,27^. The demand for goods or services can be employed by this model without being transformed into areas, and each land type can be designated a capability to supply the goods or services in need. Because the demand–supply relationships can be modeled in a many-to-many mode, CLUMondo accepts diverse demand/supply settings and allows a more realistic simulation of land changes. It has found increasing applications to simulate land change at local, regional, and global scales, as shown in Fig. 1.

In this case, the effectiveness of CLUMondo is crucial and should be improved if possible. Accordingly, this study was focused on its central mechanism, which is the transition potential of each basic unit in simulation (i.e., a cell). This transition potential is a parameter determining the future land system type of a cell. Once the transition potentials of all cells are calculated, a simulation result of CLUMondo can be immediately determined. In this study, we investigated the detailed mechanism of the transition potential. The investigation found that a key parameter in the mechanism is called conversion order, whose setting requires both expert knowledge and fine-tuning. More importantly, the values of conversion orders should vary with studies areas and land system characteristics, making the determination of conversion orders rather sophisticated. These facts are probably the reason why this key parameter should be manually set by users.

To facilitate the application of CLUMondo, we developed an automatic method for adaptively determining conversion orders. Evaluation results demonstrated that with this method, users could easily achieve a good simulation performance using CLUMondo. This method benefits not only non-expert but also expert users because its results can serve as a good starting point for fine-tuning conversion orders. We modified the source code of CLUMondo to integrate the proposed method as an option for users (who can still set conversion orders manually). To distinguish the modified CLUMondo from the official version, we referred to this modified one as CLUMondo-BNU v1.0 (where the abbreviation “BNU” stands for the university of the authors of this paper) and also released it for public use.

## CLUMondo: simulating land system changes with many-to-many demand–supply relationships

Before explaining the mechanism of CLUMondo, we introduce two common concepts in the literature on CLUMondo e.g.^28–30^, namely land system and land system services. In the context of CLUMondo literature, the concept of land system is synonymous with but broader than that of land use/cover. A land system can be simply a type of land use/cover; it can also represent a mixed type of land use/cover. In the latter complex case, land systems are defined “in terms of their land cover composition as well as land use intensity”^20^. For example, the land systems established by Jin, Jiang, Ma and Li^27^ include low/medium/high-covered natural grassland, low/medium/high-covered grassland with few livestock, low/medium/high-covered grassland with bovines, goats & sheep, extensive cropland, intensive cropland, sparse forest, and dense forest. As an extension to the concept of the land system, the concept of land system service was developed in parallel with that of ecosystem service; it refers to the area of specific land use/cover contained by a land system, or more generally, the goods or services that a land system provides for humans^31^, e.g., various terrestrial ecosystem services.

CLUMondo simulates the changes of land systems in a predefined time step, which is usually one year. Each time step involves a large number of iterations, where the default maximum number is 20,000. In the $i$-th iteration of the $t$-th time step ($i,t\ge 1$), CLUMondo determines whether and to what the land system type of every cell is changed as follows:

1 $$\mathrm{T}\left(c,t,i\right)=\left\{\begin{array}{l}\begin{array}{ll}\mathrm{T}\left(c,t,0\right) & c\in\Phi \\ \mathrm{T}\left(c,t,0\right) & \xi \left(\mathrm{T}\left(c,t,0\right)\right)<\tau \left(\mathrm{T}\left(c,t,0\right)\right) \end{array}\\ \begin{array}{ll}\left\{k|{P}_{c,k}=\underset{j}{\mathrm{max}}\left({P}_{c,1},{P}_{c,2}, \cdots ,{P}_{c,j},\cdots ,{P}_{c,n}\right)\right\}& \left\{\begin{array}{l}{\rm Con(T}\left(c,t,i\right),k)=1\\ c\notin \Phi \\ \xi \left(\mathrm{T}\left(c,t,0\right)\right)\ge \tau \left(\mathrm{T}\left(c,t,0\right)\right)\end{array}\right.\\ \mathrm{T}\left(c,t,i-1\right) & \qquad else\end{array}\end{array}\right.$$

where $c$ denotes the $c$-th cell. $k$ and $j$ denote the $k$-th and $j$-th land system type, respectively ($1\le k,j\le n$). $\mathrm{T}\left(c,t,i\right)$ is land system type of $c$ at the end of the current iteration (i.e., the $i$-th iteration of the $t$-th time step). ${P}_{c,j}$ is called the transition potential of $c$ to the $j$-th land system type; in other words, ${P}_{c,j}$ is the probability of the $c$-th cell’s land system type being converted into or maintained at the $j$-th land system type. This equation contains three “if” conditions:

- The first condition is a spatial restriction, where $\Phi$ is the restricted area where land system changes are not allowed.
- The second condition is a temporal restriction. $\xi \left(\mathrm{T}\left(c,t,0\right)\right)$ calculates how many time steps (usually years) $c$ has been maintained at the initial land system type of this time step, namely $\mathrm{T}\left(c,t,0\right)$. $\tau \left(\mathrm{T}\left(c,t,0\right)\right)$ is a non-negative integer representing the minimum time steps that the land system type $\mathrm{T}\left(c,t,0\right)$ should be maintained. This condition requires that the initial land system type of this time step should be kept for a predefined number of time steps.
- The third condition is a conversion restriction. $\mathrm{Con}(\mathrm{T}\left(c,t,i\right),k)$ indicates whether the conversion from $\mathrm{T}\left(c,t,i\right)$ to $k$ is allowed according to users’ settings, where one means allowed and zero means restricted.

From Eq. (1), it can be seen that ${P}_{c,j}$ is the key component. According to the allocation procedure outlined by van Asselen and Verburg^7^, the standard determination of ${P}_{c,j}$ is a linear combination process involving three basic factors:

2 $${P}_{c,j}=\left\{\begin{array}{ll}{P\_loc}_{c,j}+{P\_res}_{\mathrm{T}\left(c,t,0\right)}+{P\_cmp}_{i,j}& if \mathrm{T}\left(c,t,0\right)=j\\ {P\_loc}_{c,j}+{P\_cmp}_{i,j}& if \mathrm{T}\left(c,t,0\right)\ne j\end{array}\right.$$

where ${P\_loc}_{c,j}$, ${P\_res}_{\mathrm{T}\left(c,t,0\right)}$, and ${P\_cmp}_{i,j}$ are referred to as local suitability, conversion resistance, and competitive advantage, respectively. The functions and determinations of these three basic factors are as follows:

- The local suitability ${P\_loc}_{c,j}$ refers to the suitability that the $j$-th land system type occurs at the $c$-th cell. According to Eq. (2), only ${P\_loc}_{c,j}$ is a spatial parameter because it varies with location (i.e., the $c$-th cell). It is by default calculated using a logistic regression based on a series of driving factors (i.e., biophysical and/or socioeconomic conditions): where ${X}_{1,c}, {X}_{2,c}, \cdots ,{X}_{m,c}$ are the values of different driving factors at the location of $c$-th cell, and ${\beta }_{1,j}, {\beta }_{2,j},\cdots ,{\beta }_{m,j}$ are coefficients. ${\beta }_{0,j}$ is a constant. The value range of ${P\_loc}_{c,j}$ is $\left(\mathrm{0,1}\right)$, where a greater value indicates higher suitability.
  3 $$\mathrm{ln}\left(\frac{{P\_loc}_{c,j}}{1-{P\_loc}_{c,j}}\right)={\beta }_{0,j}+{\beta }_{1,j}{X}_{1,c}+{\beta }_{2,j}{X}_{2,c}+\cdots +{\beta }_{m,j}{X}_{m,c}$$

- The conversion resistance ${P\_res}_{\mathrm{T}\left(c,t,0\right)}$ reflects the difficulty (e.g., cost) of converting the land system type $\mathrm{T}\left(c,t,0\right)$ to another, or equivalently, the ease of remaining unchanged for the land system type $\mathrm{T}\left(c,t,0\right)$. Note that ${P\_res}_{\mathrm{T}\left(c,t,0\right)}$ changes along with $t$ (i.e., time step) but not $i$ (i.e., iteration). The allowed value range for ${P\_res}_{\mathrm{T}\left(c,t,0\right)}$ is $\left[\mathrm{0,1}\right]$; the greater the value, the higher the difficulty, the higher probability of keeping $\mathrm{T}\left(c,t,0\right)$, and the lower probability of converting $\mathrm{T}\left(c,t,0\right)$ to $j$. In practice, the value of ${P\_res}_{\mathrm{T}\left(c,t,0\right)}$ is usually determined according to expert knowledge or historical land system changes. For the latter case, the determination can be mathematically expressed as follows:where $c{^{\prime}}$ denotes the $c{^{\prime}}$-th cell. $h1$ and $h2$ denote two historical years, and $h1<h2$.
  4 $${P\_res}_{\mathrm{T}\left(c,t,0\right)}=\sum_{c{^{\prime}}}{y}_{c{^{\prime}},\mathrm{T}\left(c,t,0\right)}^{h1,h2}/\sum_{c{^{\prime}}}{y}_{c{^{\prime}},\mathrm{T}\left(c,t,0\right)}^{h1}$$
  5 $${y}_{c{^{\prime}},\mathrm{T}\left(c,t,0\right)}^{h1,h2}=\left\{\begin{array}{cc}1& if \mathrm{T}\left(c{^{\prime}},h\mathrm{2,0}\right)=\mathrm{T}\left(c{^{\prime}},h\mathrm{1,0}\right)=\mathrm{T}\left(c,t,0\right)\\ 0& else\end{array}\right.$$
  $${y}_{c\mathrm{^{\prime}},\mathrm{T}\left(c,t,0\right)}^{h1}=\left\{\begin{array}{cc}1& if\mathrm{ T}\left(c\mathrm{^{\prime}},h\mathrm{1,0}\right)=\mathrm{T}\left(c,t,0\right)\\ 0& else\end{array}\right.$$

- The competitive advantage ${P\_cmp}_{i,j}$ characterizes the capability of $j$, relative to other land system types, for filling the gap between the aggregated demand for land system services and the corresponding supply in the $i$-th iteration. According to van Vliet and Verburg^20^, ${P\_cmp}_{i,j}$ has the following properties:where ${P\_cmp}_{i,j,d}$ is the ${P\_cmp}_{i,j}$ specified for the $d$-th kind of land system service, ${S}_{j,d}$ is the capability of the $j$-th land system type to supply the $d$-th kind of land system service, and ${d}_{i,d}$ is the gap in the supply of the $d$-th kind of land system service.
  7 $$\left\{\begin{array}{c}{P\_cmp}_{i,j}=\sum_{d}{P\_cmp}_{i,j,d}\\ {P\_cmp}_{i,j,d}\propto {S}_{j,d},{d}_{i,d}\end{array}\right.$$

## Investigated mechanism and novel method

### Detailed mechanism of the competitive advantage

In this study, we investigated the detailed mechanism of the competitive advantage (${P\_cmp}_{i,j}$) by exploring and testing the source code for CLUMondo (https://github.com/vueg/clumondo). The detailed mechanism is mathematically expressed in this study as Eqs. (8)–(10).

8 $${P\_cmp}_{\mathrm{T}\left(c,t,0\right),j,\left(t,i\right)}={\sum }_{d}\mathrm{sign}\left({L}_{j,d}-{L}_{\mathrm{T}\left(c,t,0\right),d}\right)\cdot {\omega }_{d}\cdot {diff}_{d,\left(t,i\right)}$$

where ${L}_{j,d}$ and ${L}_{\mathrm{T}\left(c,t,0\right),d}$ are the so-called “conversion orders” of the land system types $j$ and $\mathrm{T}\left(c,t,0\right)$ when supplying the $d$-th land system service, respectively. The values of a conversion order can be $-1, 0, 1, 2, \cdots$. The greater conversion order a land system type is assigned against a land system service, the higher priority the land system type will be given in allocation for filling the gap between the demand and supply of the land system service. In particular, the value of $-1$ denotes that a land system type is of no use in filling the gap. $\mathrm{sign}\left(x-y\right)$ is a sign function (also called signum function); it returns 1 if $x>y$, 0 if $x=y$, and $-1$ if $x<y$. ${\omega }_{d}$ is a weight parameter indicating the importance of the $d$-th land system service. The greater value (with 1 as the default value) ${\omega }_{d}$ has, the more important the $d$-th land system service is.

The parameter ${diff}_{d,\left(t,i\right)}$ in Eq. (8) can be intuitively understood as the gap between the demand and supply of the $d$-th land system service in the $i$-th iteration of the $t$-th time step. However, its calculation in CLUMondo is more complex than this intuition, as shown in Eqs. (9)–(10).

9 $${diff}_{d,\left(t,i\right)}=\left\{\begin{array}{l}\begin{array}{ll}0 & \qquad i=1\end{array}\\ \begin{array}{ll}{diff}_{d,\left(t,i-1\right)}-\left(\frac{{Supply}_{d,\left(t,i-1\right)}-{Demand}_{d,t}}{{Demand}_{d,t}}\right)/\left({Speed}_{i}\times {R}_{i}\right)& i\ge 2\end{array}\end{array}\right.$$

10 $${Speed}_{i}=\left\{\begin{array}{ll}0.05 & i=1\\ {Speed}_{i-1}+0.0002& i\ge 2\end{array}\right.$$

where ${Demand}_{d,t}$ is the demand for the $d$-th land system service at the beginning of the $t$-th time step. ${Supply}_{d,\left(t,i-1\right)}$ is the supply of the $d$-th land system service by all land systems at the end of the $\left(i-1\right)$-th iteration within the $t$-th time step. According to Eq. (9), the value of ${diff}_{d,\left(t,i\right)}$ increases if ${Demand}_{d,t}>{Supply}_{d,\left(t,i-1\right)}$, whereas it decreases if ${Demand}_{d,t}<{Supply}_{d,\left(t,i-1\right)}$. ${Speed}_{i}$ and ${R}_{i}$ are two dynamic variables changing along with the iteration process to accelerate its convergence, using the following convergence conditions:

11 $$if\, \, i>\mathrm{20,000} or \left\{\begin{array}{c}\sum_{d}\frac{{Supply}_{d,\left(t,i-1\right)}-{Demand}_{d,t}}{{Demand}_{d,t}}/{n}_{d}<0.5\%\\ \frac{{Supply}_{d,\left(t,i-1\right)}-{Demand}_{d,t}}{{Demand}_{d,t}}<1\%,\forall d\end{array}\right.$$

where ${n}_{d}$ is the total number of land system services. By investigating the source code of CLUMondo, we found that ${Speed}_{i}$ had been set with an initial value of $0.05$ and an increment of 0.0002 per iteration. We also found that ${R}_{i}$ is a random number ranging from 322 to 365. The incorporation of ${Speed}_{i}$ and ${R}_{i}$ gradually reduces the amount of change in ${diff}_{d,\left(t,i\right)}$ along with the iteration, further making the number of cells to be changed smaller and smaller in each iteration. This decreasing number facilitates the convergence of the iteration when minor changes to land systems are needed.

### Difficulty in the manual setting of conversion orders

Having understood the detailed mechanism of the competitive advantage, one may realize the important role of the conversion orders therein and the importance of their determination. This is probably why the determination should be performed manually and carefully in CLUMondo. van Asselen and Verburg^7^ illustrated one such determination, with the result shown in Table 1. On the explanation of this table, van Asselen and Verburg^7^ noted that it “indicates the relative order of the land systems contribution to fulfilling a specific demand type” and also “ensures logical trajectories of land change” (p. 3651). They recommended determining conversion orders “differently by region, depending on the land system characteristics in the specific regions and the likely trajectories of fulfilling increasing (or decreasing) demands” (p. 3651), implying that the determination is sophisticated and requires fine tuning.

**Table 1:** Capability and conversion orders determined by van Asselen and Verburg^7^ for 30 different land systems in supplying four defined land system services: crop production (tons), land-based livestock (bovines, goats, and sheep; number), landless livestock (pigs and poultry; number), and built-up area (km^2^).

	Crop production (tons)		Land-based livestock (number)		Landless livestock (number)		Built-up area (km^2^)	
	Capability	Order	Capability	Order	Capability	Order	Capability	Order
Cropland extensive, few livestock (ls.)	8977	4	4658	2	9620	2	0.11	1
Cropland extensive, land-based ls	11,047	4	10,250	3	31,630	2	0.12	1
Cropland extensive, landless ls	11,110	4	3317	2	81,968	3	0.11	1
Cropland med. intensive, few ls	11,695	5	3704	2	11,899	2	0.36	1
Cropland med. intensive, land-based ls	13,421	5	14,282	4	79,960	2	0.40	1
Cropland med. intensive, landless ls	16,363	5	4387	2	102,894	4	0.43	1
Cropland intensive, few ls	24,076	6	2076	2	7934	2	0.69	1
Cropland intensive, land-based ls	37,740	6	23,949	5	339,985	5	1.24	1
Cropland intensive, landless ls	31,785	6	4264	2	172,779	5	0.67	1
Mosaic cropland and grassland, land-based ls	13,563	4	13,843	4	132,327	4	0.52	1
Mosaic cropland and grassland, landless ls	16,080	4	4005	2	122,532	4	0.46	1
Mosaic cropland (ext.) and grassland, few ls	3871	2	4736	2	8152	2	0.09	1
Mosaic cropland (med.) and grassland, few ls	6504	3	4403	2	11,890	2	0.25	1
Mosaic cropland (int.) and grassland, few ls	10,984	4	3374	2	8815	2	0.47	1
Mosaic cropland and forest, landless ls	14,548	3	3815	2	112,431	4	0.33	1
Mosaic cropland (ext.) and open forest, few ls	6,104	2	3754	2	15,727	2	0.09	1
Mosaic cropland (med. int.) and forest, few ls	6752	3	3511	2	13,386	2	0.17	1
Mosaic cropland (intensive) and forest, few ls	9774	4	3127	2	12,680	2	0.32	1
Dense forest	1478	1	2368	$-$ 1	28,849	$-$ 1	0.07	1
Open forest, few ls	1459	1	2302	1	8976	1	0.09	1
Open forest, landless ls	4576	$-$ 1	3073	$-$ 1	89,483	3	0.13	1
Mosaic grassland and forest	3043	1	3441	1	38,943	1	0.14	1
Mosaic grassland and bare	381	1	2824	1	3294	1	0.08	1
Natural grassland	749	1	0	1	0	1	0	1
Grassland, few ls	1610	1	2720	1	18,250	1	0.13	1
Grassland, land-based ls	2059	$-$ 1	14,159	4	37,991	$-$ 1	0.23	1
Bare	18	$-$ 1	4	$-$ 1	4	$-$ 1	0	1
Bare, few ls	430	$-$ 1	2928	1	2948	1	0.04	1
Peri-urban and villages	22,056	$-$ 1	9110	$-$ 1	184,526	$-$ 1	8.97	2
Urban	17,796	$-$ 1	5010	$-$ 1	193,283	$-$ 1	37.60	3

*ls.* Livestock, *ext.* extensive, *med.* medium intensive, *int.* intensive.

We recognize the necessity for manually determining the conversion orders, but we note the difficulty in the determination by non-expert users, especially beginners. To overcome the difficulty, we will propose an automatic method for adaptively determining the conversion orders for different land systems. This automatic method will be incorporated into CLUMondo as an option for non-expert users, as well as for expert users to find a good starting point for fine-tuning.

### A method for automatically determining conversion orders

This study presents a method for automatically determining the conversion orders of different land systems based on their capability for supplying a specific service. The method is powerful in that it is effective in improving the simulation accuracy of CLUMondo, efficient in operation, and widely applicable.

Before developing the method, we rethink the functionality of the conversion order as a parameter of the competitive advantage. As noted in Section “Detailed mechanism of the competitive advantage”, the conversion order was initially not included as a parameter of the competitive advantage, which was designed to be proportional to ${S}_{j,d}$ (the capability of the $j$-th land system type to supply the $d$-th kind of land system service) in concept. However, as shown in Section “Difficulty in the manual setting of conversion orders”, the conversion order was included in implementing CLUMondo, whereas ${S}_{j,d}$ is not used in practice. The conversion order is employed as a proxy of ${S}_{j,d}$, to avoid the competition in filling the demand–supply gap of a land system service between two land systems with similar capabilities for supplying that service. For example, as shown in Table 1, the “extensive cropland system with few livestock” was assigned the same conversion order (i.e., 1) as the “intensive cropland system with few livestock” in order not to promote the conversion from the former type to the latter type when filling the demand–supply gap of the “built-up area” service, although the former type has a lower capability in supplying the “built-up area” service than the later type (i.e., 0.11 vs. 0.69). Essentially, this functionality of the conversion order is achieved by transforming ${\left\{{S}_{j,d}\right\}}_{j}$ from a series of ratio values to categorized, ordinal ones refer to^32^ for the nominal, ordinal, interval, and ratio scales of measurement.

**Figure 2:**
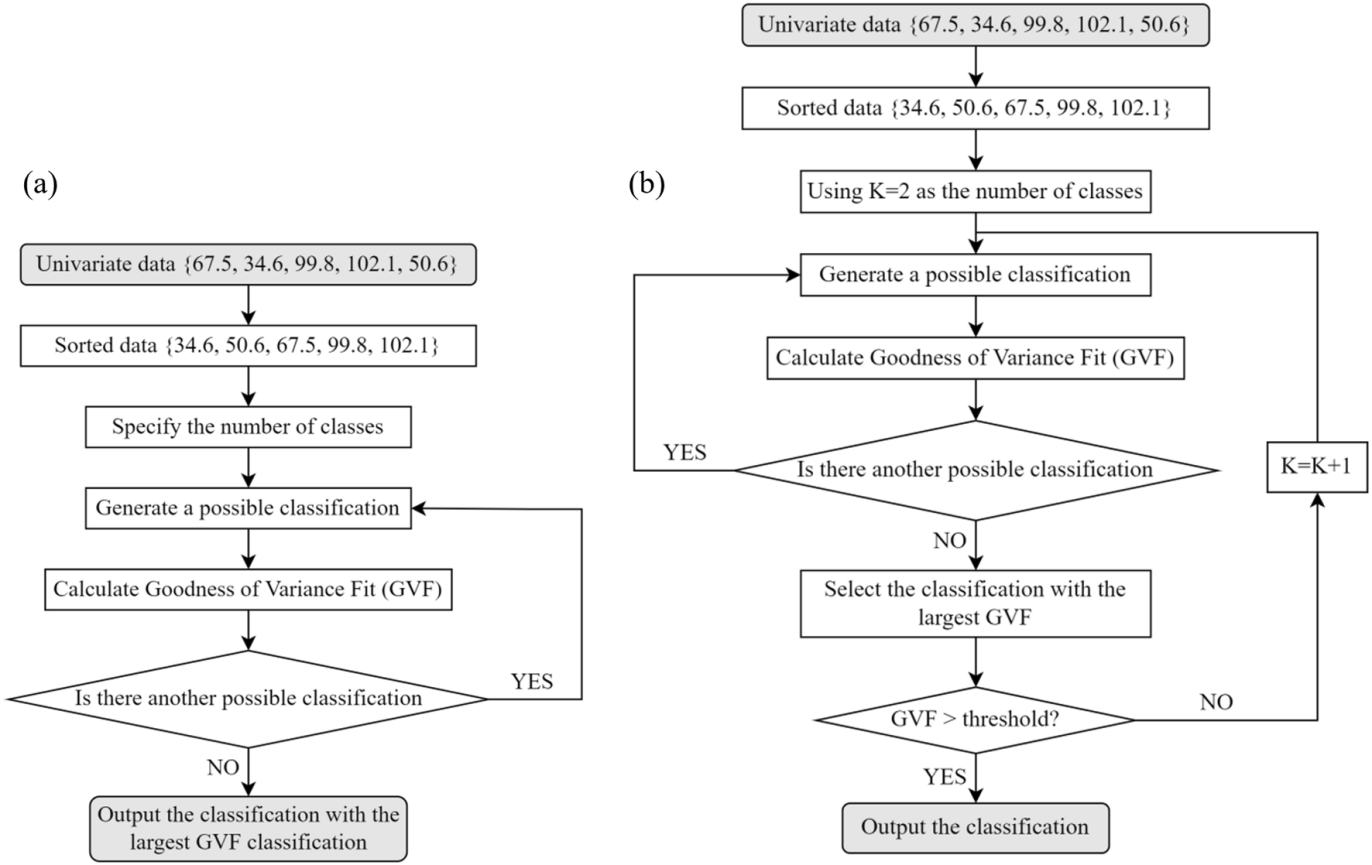
General algorithm of Natural Breaks (**a**) and the modified version proposed in this study (**b**).

From this understanding of the functionality, we propose to automatically determine the conversion orders of different land systems using the classification of univariate data. To this end, we adopted the time-tested and overwhelmingly popular classification algorithm for univariate data, namely Natural Breaks^33–35^. Natural Breaks is to find a classification of univariate data by maximizing the total difference between every two classes and minimizing the total difference within each class. The general algorithm of Natural Breaks is an enumeration of all possible classifications (Fig. 2a), from which the one with the largest goodness of variance fit [GVF, Eq. (12)] is selected.

12 $$GVF=1-\frac{\mathrm{SDCM}}{\mathrm{SDAM}}=1-\sum_{x}\sum_{y}{\left({Z}_{x,y}-{M}_{x}\right)}^{2}/\sum_{x}\sum_{y}{\left({Z}_{x,y}-M\right)}^{2}$$

where $\mathrm{SDCM}$ denotes the sum of squared deviations from the class means, and $\mathrm{SDAM}$ denotes the sum of squared deviations from the array mean (here, the array means all values of the univariate data). ${Z}_{x,y}$ is the $y$-th value in the $x$-th class, ${M}_{x}$ is the mean of all values in the $x$-th class, and $M$ is the mean of all values in all classes.

However, there is a practical problem in adopting Natural Breaks. As shown in Fig. 2a, Natural Breaks works with a user-specified number of classes. In the case of CLUMondo, this number should not be static and should be capable of varying with different applications, or more specifically, with different sets of ${\left\{{S}_{j,d}\right\}}_{j}$. A straightforward approach to address this problem is to slightly alter the algorithm to make it perform a complete enumeration. The so-called complete enumeration aims to select the classification scheme with the largest GVF by enumerating all possible classification schemes under all possible numbers of classes. But such a straightforward approach is infeasible for two reasons. First, this approach is inefficient as it substantially increases the number of possibilities. Second and more important, the largest GVF in theory (i.e., 1 when $\mathrm{SDCM}=0$) will be achieved only if the number of classes equals the total number of values, meaning that there is no classification at all.

In this study, we propose to solve the preceding problem by modifying the Natural Breaks algorithm. Our core idea is to incorporate a threshold of GVF into the algorithm to stop complete enumerations. In this way, users no longer need to specify the number of classes; more importantly, this number will be adaptively determined. Specifically, the modified algorithm iterates all possible numbers of classes, i.e., from the smallest (i.e., 2) to the largest one (i.e., the total number of values). Within each iteration (i.e., under each number of classes), the modified algorithm further enumerates all possible classification schemes. Note that different classification schemes have the same number of classes at this stage. Each classification scheme corresponds to a GVF. A comparison will be made between the largest GVF observed and the threshold. If that GVF is greater, then the enumeration will be stopped, and the classification scheme corresponding to that GVF will finally be adopted. Otherwise, the number of classes will be increased by one to start the next iteration. The workflow of the modified algorithm is summarized in Fig. 2b.

In practically utilizing this modified algorithm of Natural Breaks, we set the threshold of GVF as 0.8, which is an empirical value e.g.^36^ and indicates an excellent classification. To automatically determine the conversion orders of different land systems in supplying the $d$-th service, we apply the modified algorithm to ${\left\{{S}_{j,d}\right\}}_{j}$ and obtain a resultant classification scheme as follows: $\left\{{\Phi }_{1}<{\Phi }_{2}<\cdots <{\Phi }_{\rm K}\right\}$, where ${\rm K}$ is the number of classes, and ${\Phi }_{\vartheta 1}<{\Phi }_{\vartheta 2}$ means that the average of the $\vartheta 1$-th class is smaller than that of the $\vartheta 2$-th class. This resultant classification scheme is translated into conversion orders according to the following mapping: ${L}_{j,d}=\kappa -1$ where ${S}_{j,d}\in {\Phi }_{\kappa }$.

## Experimental evaluation

### Study areas and raw data

To select study areas, we consider the following three criteria. First, the study area should not be too small to ensure the complexity of land system changes. For example, a study area of a small city is not desirable accordingly because its land system changes are probably monotonous. Second, there should be more than one study area, to avoid the coincidence of evaluation results. Ideally, study areas should have distinct structures of land use, in terms of the composition of land system types and/or their spatial patterns. The third criterion is a practical issue: data availability and sufficiency. The data used for experimental evaluation should include land system data with a fine spatial resolution for two historical years and various potential driving factors with the same spatial resolution as the land system data.

**Figure 3:**
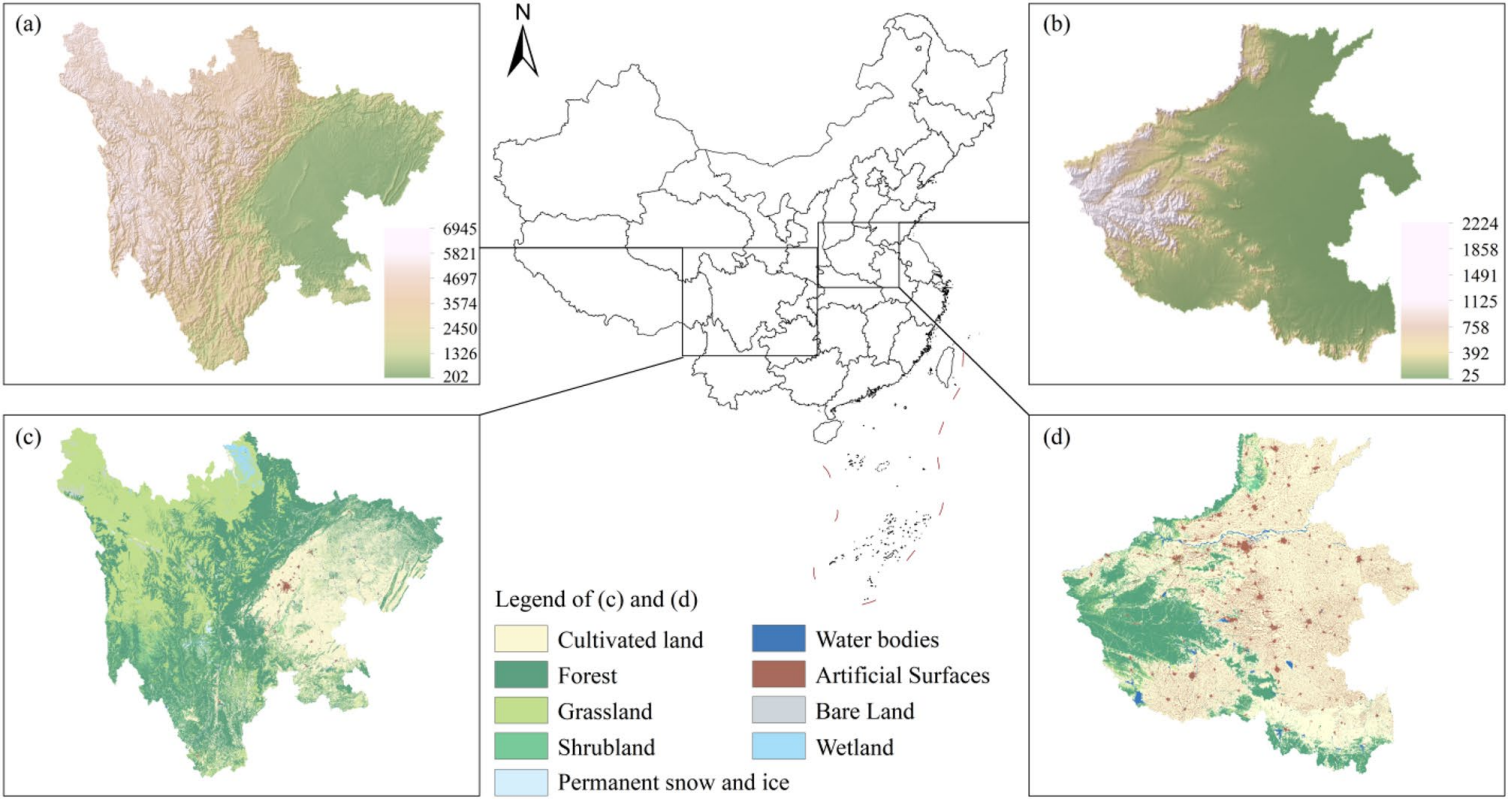
Study areas. (**a**) Topography of Sichuan; (**b**) Topography of Henan; (**c**) GlobeLand30 land use/cover of Sichuan; and (**d**) GlobeLand30 land use/cover of Henan. The land use/cover data can be obtained from the website of the GlobeLand30 (http://www.globallandcover.com/). The topography data can be obtained from the website of the WorldClim (https://www.worldclim.org/). The figure is generated using ArcGIS 10.7 (http://www.esri.com/software/arcgis/arcgis-for-desktop/).

According to the preceding criteria, we selected two study areas, namely the Sichuan and Henan provinces of China. Their geographic locations are shown in Fig. 3. Sichuan has a large area of 486,000 km^2^, ranking fifth among the 34 Chinese provinces (or equivalent administrative units). The province covers the western part of a lowland region called the Sichuan Basin, surrounded by the Himalayas to the west, the Qinling range (i.e., the Qin Mountains) to the north, and the mountainous areas of Yunnan Province to the south. The topography of Sichuan is characterized by a considerable decrease in elevation from west to east, as shown in Fig. 3a. Dominant types of land use/cover of Sichuan include forests (40.4%), grasslands (30.7%), and cultivated lands (24.1%), and the proportion of urban areas is noticeably tiny (0.49%), according to the 2010 dataset of GlobeLand30^37^.

Henan is a province in the central part of China, covering a large part of the agriculturally fertile and densely populated North China Plain. It is an agricultural province with food production of 65.4 million tons per year, ranking second out of the 34 Chinese provinces or equivalent administrative units^38^. The population of Henan is 99.3 million^39^, which ranks third in China and is greater than 94% of countries (or dependent territories) according to the data by the United Nations^40^. In comparison to Sichuan, the topography of Henan is dominated by a flat plain with a few highlands, as shown in Fig. 3b. In addition, the structure of land use/cover in Henan is quite different from Sichuan. Cultivated lands, forests, and urban areas are the first three major types of land use/cover, occupying 64.9%, 19.4%, and 11.3% of Henan’s total area, respectively.

The experimental evaluation relies on two types of raw data: land use/cover data and potential driving factors. This study set two criteria for preparing the land use/cover data. First, the data of Sichuan and Henan should be available for at least two periods. The data for the earlier period are used as the starting point of the simulation, whereas that for a later period is used as the benchmark for the simulation results. Second, the data should have a fine spatial resolution to facilitate the generation of land systems at a coarse scale. According to the two criteria, we employed the GlobeLand30 datasets^37^, a 30-m resolution global land cover product released for 2000, 2010, and 2020. The thematic resolution of GlobeLand30 is ten types of land cover, i.e., cultivated land, forest, grassland, shrubland, wetland, water bodies, tundra, artificial surfaces, bare land, and permanent snow/ice. We extracted the 2010 and 2020 data for Sichuan and Henan from GlobeLand30. Note that the extracted GlobeLand30 are only the raw data of our 1-km resolution land system.

**Figure 4:**
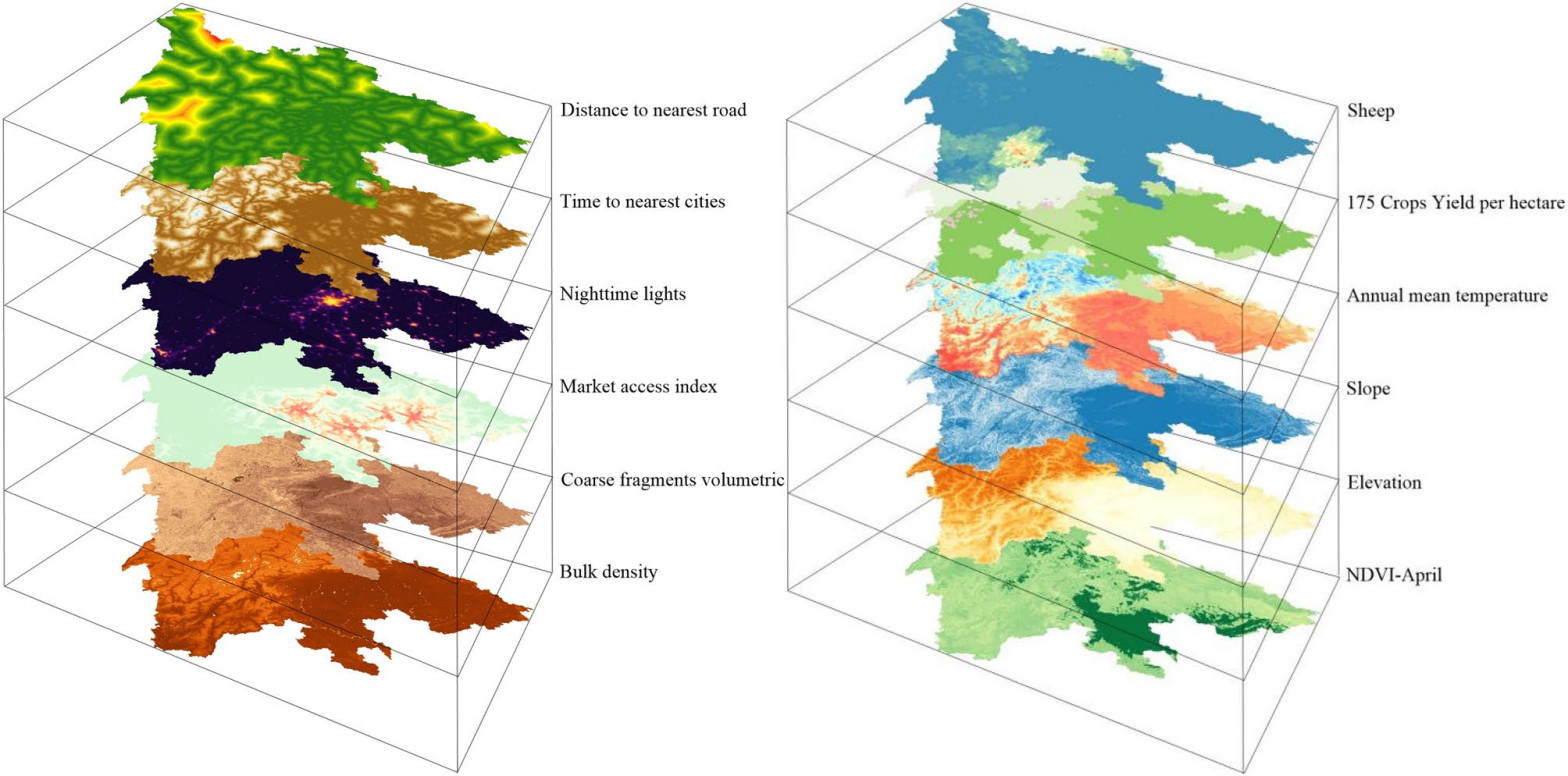
Some potential driving factors prepared for the study area of Sichuan. The data source can be found in Table 2. The figure is generated using Surfer 11 (https://www.goldensoftware.com/products/surfer).

Potential driving factors should be prepared at the same spatial resolution of the land systems and as diverse as possible. Because we will produce land system data at the spatial resolution of 1 km, the expected spatial resolution of potential driving factors is 1 km. We collected or generated a total of 55 1-km potential driving factors, which can be classified into seven categories as shown in Table 2. Some of the potential driving factors are visualized in Fig. 4.

**Table 2:** Potential driving factors.

Category	No.	Data	Year	Source
Soil	1	Bulk density	2017	^41^
	2	Cation exchange capacity	2017	
	3	Clay content	2017	
	4	Coarse fragments volumetric	2017	
	5	Derived available soil water capacity	2017	
	6	Organic carbon density	2017	
	7	pH in H_2_O	2017	
	8	Sand content	2017	
	9	Silt content	2017	
	10	Texture class	2017	
Socio-economic	11	Market access index	2011	^42^
	12	Market influence index ($/person)	2011	
	13	Market density index	2011	
	14	Night-time lights	2010	DMSP-OLS Version 4
	15	Total GDP (based on purchasing power parity)	2015	^43^
	16	Gridded population of the world	2010	^44^
Accessibility	17	Time to nearest cities	2015	^45^
	18	Distance to the nearest river	N/A	Calculated in this study
	19	Distance to the nearest road	N/A	
	20	Distance to the nearest railway	N/A	
	21	Travel time one meter (motorized)	2019	^46^
	22	Travel time one meter (walking-only)	2019	
	23	Time to the nearest healthcare facility (motorized)	2019	
	24	Time to the nearest healthcare facility (walking-only)	2019	
Agriculture and vegetation	25	175 crops yield per hectare	2000	^47^
	26	Gross primary production-March/June/September/December	2010	^48^
	27	Normalized Difference Vegetation Index (NDVI)-March	2010	10.5281/zenodo.3256275
	28	NDVI-June/September/December	2010	
Terrain	29	Elevation	2000	^49^
	30	Variance of elevation	N/A	Calculated in this study
	31	Slope	N/A	
	32	Aspect	N/A	
Climate	33	Annual mean precipitation	2007–2018 avg	10.5067/modis/myd13a2.006
	34	Mean precipitation-March/June/September/December	2007–2018 avg	
	35	Annual mean temperature	2000–2017 avg	10.5281/zenodo.1435938
	36	Mean temperature-March/June/September/December	2000–2017 avg	
Livestock	37	Buffaloes	2010	10.7910/dvn/5u8mwi
	38	Cattle	2010	10.7910/dvn/givq75
	39	Chickens	2010	10.7910/dvn/sufasb
	40	Ducks	2010	10.7910/dvn/ichcbh
	41	Goats	2010	10.7910/dvn/ocph42
	42	Horses	2010	10.7910/dvn/7q52mv
	43	Pigs	2010	10.7910/dvn/33n0jg
	44	Sheep	2010	10.7910/dvn/blwpzn

*N/A* not available, *“avg”* average (because only average data were released).

### Establishment of multifunctional land systems

As noted in the introduction, CLUMondo features the capability of simulating land changes with many-to-many demand–supply relationships. Therefore, a comprehensive evaluation should be carried out to exploit such featured capability, where the key lies in establishing multifunctional land systems. The establishment involves two steps: generating a taxonomy of land systems and quantifying the services of different land systems.

**Figure 5:**
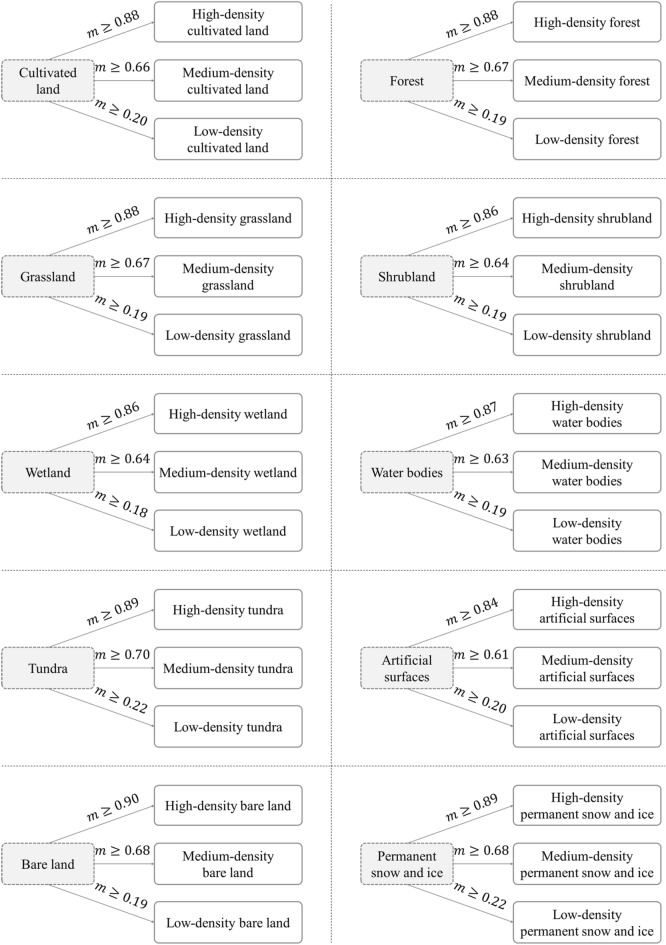
Taxonomy of land systems for our study areas (${\varvec{m}}$ denotes the threshold).

In this study, we generated the taxonomy of land systems based on the scale transformation of the GlobeLand30 datasets, or more specifically, by transforming the spatial resolution of the GlobeLand30 datasets from 30 m to 1 km. First, we upscaled the GlobeLand30 datasets from the initial spatial resolution of 30 m to a coarser resolution of 990 m, by aggregating every $33\times 33$ pixels (referred to as micro-pixels) of the raw data into new ones (referred to as macro-pixels). For more information on the aggregation technique, we refer the reader to materials on the multiscale representation of spatial data^50^. Then, we specified the land system type of each macro-pixel as the dominated type of corresponding micro-pixels, and we further distinguished three levels of dominance, namely high, medium, and low-density. Accordingly, we generated as many as 30 land systems, such as high/medium/low-density forests, as shown in Fig. 5. In particular, thresholds for the three levels of each dominated type of micro-pixel were determined using Natural Breaks with a designated classification number of three; values of these thresholds are also shown in Fig. 5. Finally, the land systems were slightly resampled to the spatial resolution of 1 km to match the resolution of most potential driving factors.

The services of each land system were defined as the area of each of the ten types of GlobeLand30 land use/cover. Under this definition, each land system would potentially become multifunctional to supply all ten services. Because our land systems were generated by transforming the spatial resolution of GlobeLand30, a pixel of any land system (a macro-pixel) corresponds to many GlobeLand30 pixels (i.e., micro-pixels) with usually diverse types. To quantify the capability of each land system in supplying every service (e.g., in 2010), we first performed an overlay analysis between the generated land systems and their corresponding raw data of land use/cover (i.e., the 2010 dataset of GlobeLand30). Based on the resultant overlaps, the capability can be determined using the following equation:

13 $${S}_{j,d}={\Lambda }_{j,d}/{\Lambda }_{j}$$

where ${\Lambda }_{j}$ denotes the total area of the $j$-th land system in a given study area, and ${\Lambda }_{j,d}$ is the total area of the micro-pixels that overlap the $j$-th land system and have the $d$-th type of GlobeLand30 land use/cover (i.e., the $d$-th service). The units of ${S}_{j,d}$ are ${\text{km}}^{2}/{\text{km}}^{2}$. The aggregated demand for the $d$-th service in a year (e.g., 2020) was calculated as the total area of the $d$-th type of pixel (i.e., micro-pixels) in the GlobeLand30 dataset of that year.

### Settings of other simulation parameters

In addition to establishing multifunctional land systems, some other parameters must be set before running CLUMondo, such as local suitability and conversion resistance. Since these parameters were not the objective of our experimental evaluation, we adopted default but reasonable settings, or setting methods, if possible.

The location suitability was calculated using the default method, i.e., the logistic regression based on a series of driving factors, as shown in Eq. (3). Note that not all of our potential driving factors (as previously shown in Table 2) were included in the logistic regression. We removed some potential driving factors to reduce the correlation among them. Specifically, we first measured the correlation between each pair of potential driving factors using Spearman’s rank correlation coefficient (SRCC). Then, for each pair with an SRCC greater than 0.9, we removed from the pair the one that is more correlated with all other potential driving factors. To determine which is more correlated with others, we calculated the sum of SRCCs between the one potential driving factor and each other. Third, the first two steps were repeated until the SRCC of each pair of potential driving factors were less than 0.9. It is also noted that not all spatial locations within the study area were included in the logistic regression. We sampled the study area using an interval of one pixel; thus, only approximately 25% of locations were used for regression. Such a sampling strategy avoids the selection of neighboring locations, so it improves the independence of our samples.

The conversion resistance of each land system, ${P\_res}_{\mathrm{T}\left(c,t,0\right)}$, was determined using historical land system changes with Eqs. (4)–(6), where $h1=2010$ and $h2=2020$. The value of $\mathrm{Con}(\mathrm{T}\left(c,t,i\right),k)$ was set by checking whether these are conversions from the land system type of $\mathrm{T}\left(c,t,i\right)$ in 2010 to the $k$-th land system type $in 2020$. To avoid noise, we introduced a threshold of 1% in the check. Only when the area of conversion is larger than 1% of the study area, the value $\mathrm{Con}(\mathrm{T}\left(c,t,i\right),k)$ was set as 1; otherwise, it was set as 0. In the experimental evaluation, we do not employ spatial and temporal restrictions, which are optional in Eq. (1).

### Benchmarks and evaluation metrics

We have both comparative and benchmark experiments. These two categories of experiments shared the same experimental settings for a study area but different determinations of conversion orders. In the comparative experiments, conversion orders were determined using the automatic method proposed in this study. By contrast, in the benchmark experiments, conversion orders were determined objectively according to the capabilities of different land systems to supply services and also by ensuring their reflection of “the relative order of the land systems contribution to fulfilling a specific demand type”^7^, as follows:

14 $${L}_{j,d}=\left\{\begin{array}{cc}-1 & {S}_{j,d}=0\\ Rank\left({S}_{j,d}\right)& {S}_{j,d}\ne 0\end{array}\right.$$

where $Rank\left({S}_{j,d}\right)$ returns the order (starting from 1) of ${S}_{j,d}$ in the ascending sequence of ${\left\{{S}_{j,d}\ne 0\right\}}_{d}$.

To evaluate the performance of our logistic regressions, we drew receiver operating characteristic (ROC)^51^ curves to assess the fit of the logistic regression established for each land system. We employed a measure developed with the ROC curves to quantify each regression’s goodness of fit: Area Under the Curve (AUC, sometimes referred to as the ROC value)^52^. The theoretical value of AUC ranges from 0.5 to 1, where a higher value indicates a better fit. According to expert experience e.g.,^22,27,53^, an AUC value of 0.7 or above means good fit, and that of 0.9 or above demonstrates excellent fit.

We utilized two popular metrics to evaluate the performance of land change simulation: the standard Kappa index of agreement and the total disagreement. The former metric is also called the Kappa statistic^54^ or the Kappa index^23^. It is an improved measure compared with fraction correct (also called proportion correct or proportion agreement), which is biased in most cases when applied to land system maps with unevenly distributed categories of cells. Its calculation incorporates the expected proportion of agreement due to chance, as follows:

14 $$Kappa=\left({P}_{0}-{P}_{e}\right)/\left(100\%-{P}_{e}\right)$$

where ${P}_{0}$ is the proportion of agreement calculated between the simulated and the actual land systems in 2020, and ${P}_{e}$ is the expected proportion of agreement due to chance. The Kappa statistic is a positive metric: The greater the Kappa statistic, the better the performance of a land change simulation.

The latter metric ($D$) was proposed by Pontius Jr and Millones^55^ as an alternative to the Kappa statistic, as follows:

15 $$\left\{\begin{array}{l}D=0.5*\left(\sum_{g=1}^{J}{q}_{g}+\sum_{g=1}^{J}{a}_{g}\right)\\ {q}_{g}=\left|\sum_{i=1}^{J}{p}_{ig}-\sum_{j=1}^{J}{p}_{gj}\right|\\ {a}_{g}=2*min\left[\begin{array}{ll}\left(\sum_{i=1}^{J}{p}_{ig}\right)-{p}_{gg},& \left(\sum_{j=1}^{J}{p}_{gj}\right)-{p}_{gg}\end{array}\right]\end{array}\right.$$

where $J$ is the total number of land system types, and ${p}_{ij}$ is the proportion of the study area that is of $i$-th land system type in the simulation result and the $j$-th land system type in the reference result. The total disagreement is a negative metric: The smaller the total disagreement, the better the performance of a land change simulation.

### Results and analysis

The evaluation results of our logistic regressions are shown in Table 3, which consists of the AUCs of all logistic regressions established for each land system of the two study areas. For the study area of Sichuan, we can see from this table that all AUCs are greater than 0.700 and averaged at 0.913. The proportion of AUCs greater than 0.900 is 66% (18 out of 27), and that of AUCs greater than 0.800 is as high as 89% (24 out of 27). These results demonstrate that our incorporation of a large number of diverse, potential driving factors into logistic regression is valid and highly effective. These results also demonstrate the excellent fit of the vast majority of the established logistic regressions. We also noticed from Table 3 the following pattern: the AUC generally decreases from a high-density land system to the corresponding medium-density land system and then the low-density one. This pattern makes sense because the cell-level heterogeneity reduces from a high-density land system to the corresponding medium-density and low-density ones. The higher cell-level heterogeneity a land system has, the more significant relationship can be established between the land system and its driving factors.

**Table 3:** Evaluation results (AUCs) of logistic regressions in the study area of Sichuan.

ID	Land system type	AUC
0	Low-density cultivated land	0.856
1	Medium-density cultivated land	0.891
2	High-density cultivated land	0.964
3	Low-density forest	0.730
4	Medium-density forest	0.756
5	High-density forest	0.908
6	Low-density grassland	0.730
7	Medium-density grassland	0.802
8	High-density grassland	0.945
9	Low-density shrubland	0.907
10	Medium-density shrubland	0.924
11	High-density shrubland	0.911
12	Low-density wetland	0.964
13	Medium-density wetland	0.975
14	High-density wetland	0.999
15	Low-density water surfaces	0.889
16	Medium-density water surfaces	0.822
17	High-density water surfaces	0.864
18	Low-density artificial surfaces	0.964
19	Medium-density artificial surfaces	0.981
20	High-density artificial surfaces	0.999
21	Low-density bare land	0.918
22	Medium-density bare land	0.977
23	High-density bare land	0.994
24	Low-density permanent snow and ice	0.987
25	Medium-density permanent snow and ice	0.992
26	High-density permanent snow and ice	0.999

Similar findings can be made for the study area of Henan. As shown in Table 4, more than half (61%, 14 out of 23) of Henan’s AUCs have a value greater than 0.950. The proportion of AUCs greater than 0.900 reached 78% (18 out of 23), and that of AUCs greater than 0.800 was 87% (20 out of 23). The average of all AUCs is 0.928, which is even greater than that of Sichuan’s AUCs. Therefore, our logistic regressions for Henan are also valid and highly effective.

**Table 4:** Evaluation results (AUCs) of logistic regressions in the study area of Henan.

ID	Land system type	AUC
0	Low-density cultivated land	0.658
1	Medium-density cultivated land	0.715
2	High-density cultivated land	0.776
3	Low-density forest	0.877
4	Medium-density forest	0.903
5	High-density forest	0.982
6	Low-density grassland	0.905
7	Medium-density grassland	0.918
8	High-density grassland	0.965
9	Low-density shrubland	0.998
10	Medium-density shrubland	0.997
11	High-density shrubland	0.995
12	Low-density wetland	0.994
13	Medium-density wetland	0.937
14	High-density wetland	0.963
15	Low-density water surfaces	0.955
16	Medium-density water surfaces	0.980
17	High-density water surfaces	0.997
18	Low-density artificial surfaces	0.846
19	Medium-density artificial surfaces	0.918
20	High-density artificial surfaces	0.991
21	Low-density bare land	0.992
22	Medium-density bare land	0.989
23	High-density bare land	–

**Figure 6:**
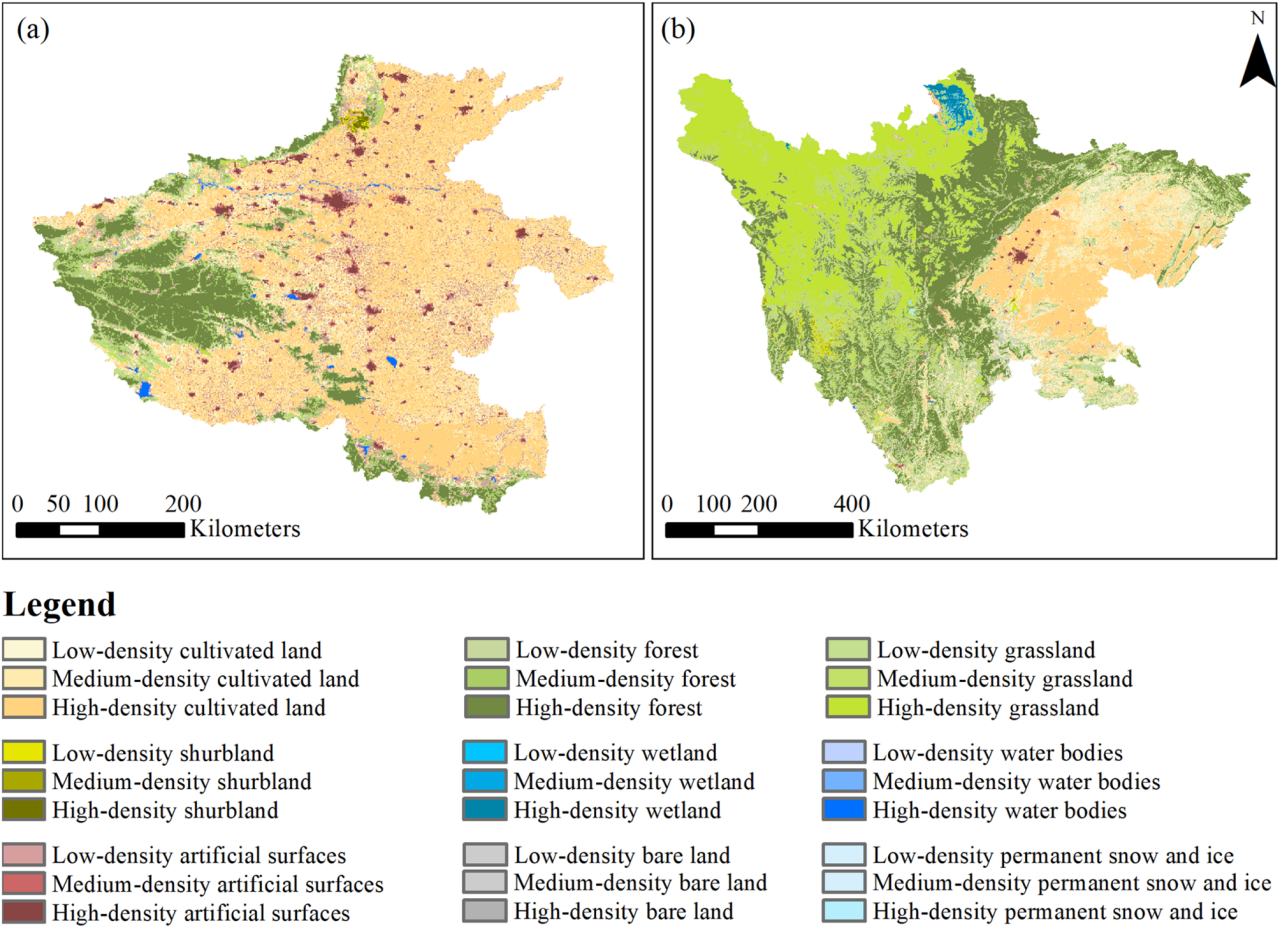
Land change simulation results of Henan (**a**) and Sichuan (**b**). The data were generated in this study. The figure is generated using ArcGIS 10.7 (http://www.esri.com/software/arcgis/arcgis-for-desktop).

The evaluation results of our land change simulations in the two study areas are shown in Fig. 6 and Table 5. It can be seen from this table that for the study area of Sichuan, the Kappa statistics of its benchmark and comparative experiments are 0.4287 and 0.8656, respectively. Thus, the Kappa statistic obtained in the comparative experiment increased by 101.91% compared to that in the benchmark experiment. This considerable increase demonstrates that the proposed method is highly effective. Similar conclusions can be drawn from the total disagreement. The total disagreement of the benchmark experiment is 0.5047, whereas that of the comparative experiment was reduced substantially to 0.1169 (with a reduction rate of 76.84%). For the study area of Henan, both the Kappa statistic and the total disagreement demonstrated the effectiveness of the proposed method. Specifically, the Kappa statistic was increased from 0.6475 in the benchmark experiment to 0.6823 in the comparative experiment, and the total disagreement was decreased from 0.2824 to 0.2535.

**Table 5:** Evaluation results of our land change simulations.

Study area	Experiment	Kappa	Total disagreement
Sichuan	Benchmark	0.4287	0.5047
	Comparative	0.8656	0.1169
Henan	Benchmark	0.6475	0.2824
	Comparative	0.6823	0.2535

Overall, our evaluation results with the two study areas demonstrate not only the effectiveness of the proposed method for adaptively determining conversion orders but also the method’s high applicability. The method is especially of use if the simulation performance of CLUMondo is poor (e.g., with the study area of Sichuan).

## Discussion

In the proposed method for adaptively determining conversion orders, we adopted an empirical value of GVF (= 0.8) in adopting our modified algorithm of Natural Breaks. To test the effectiveness of this empirical value, we performed further experiments in the two study areas. In these experiments, we first employed the general algorithm of Natural Breaks instead of our modified algorithm. In utilizing the general algorithm, we enumerated and tried every possible number of classes. This number ranged from 1 to 27 with the study area of Sichuan (as Sichuan has 27 land systems), and it went from 1 to 24 with the study area of Henan (as Henan has 24 land systems). Each possible number of classes results in a unique classification of the capabilities of different land systems (i.e., ${L}_{j,d}$). Then, each classification was translated into a unique set of conversion orders using the same method of the modified algorithm, namely ${L}_{j,d}=\kappa -1$ where ${S}_{j,d}\in {\Phi }_{\kappa }$. We performed independent experiments with each set of conversion orders (i.e., each possible number of classes).

**Figure 7:**
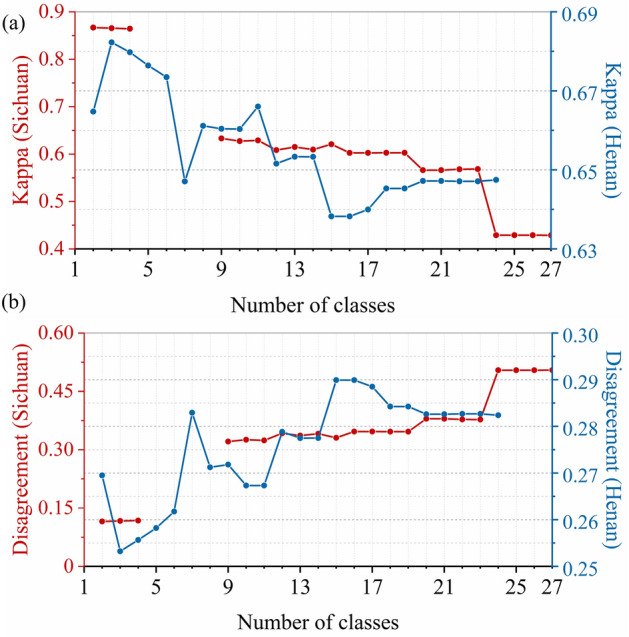
Evaluation results with each possible number of classes: (**a**) the Kappa statistic and (**b**) the total disagreement.

The evaluation results of each experiment are shown in Fig. 7. For both study areas, no simulation results (and thus no evaluation results) were obtained when the number of classes equaled one (i.e., when all land systems have the same conversion orders). This fact justified the importance of conversion orders and the necessity of studying how to determine them. There are also some other cases where the evaluation results were not obtained. These cases were due to the failure of CLUMondo to produce a simulation result and thus excluded from our analysis. For the experiments where the evaluation results were available, we have the following two findings:

- For the study area of Sichuan, the Kappa statistic researched its highest level (i.e., greater than 86%) when the number of classes is small (i.e., 2–4). Then, the Kappa statistic underwent a decreasing trend with more classes, and a sharp decrease can be observed when the number of classes was increased from 23 to 24. When the number of classes equalled or exceeded 24, the Kappa statistic would become smaller than 45%. The trend shown by the values of total disagreement is opposite to that of the Kappa statistic in this case. We further calculated the correlation between these two sets of values, finding that their SRCC is as high as – 0.999.
- For the study area of Henan, the Kappa statistic and the total disagreement witnessed a general downward trend and a general upward trend along with the increase of the number of classes, respectively. The SRCC between the two metrics is – 0.992 in this case. Our proposed method corresponds to the experiment in which the number of classes is three. It can be seen the proposed method led to the best performance.

Based on these two findings, we concluded that the empirical value of GVF (= 0.8) is effective and an excellent choice. It is effective because its resultant values of the Kappa statistic are among the highest ones for both study areas. In addition, it is an excellent choice as it should no longer be increased (or decreased), especially with the study area of Henan.

Our proposed method facilitates applications of CLUMondo with complex land systems (in terms of not only the number of land use/cover types but also the consideration of land use intensity). As noted in the introduction section, CLUMondo is becoming increasingly popular. But recent applications of CLUMondo still rely on simple land systems, which include only several land use/cover types. Let us take some recent studies as examples. Wang et al.^56^ performed a cost–benefit analysis of China’s forest landscape restoration policy with CLUMondo simulations involving six land use/cover types. While assessing the impact of global initiatives on land restoration scenarios in India, Edrisi et al. simulated the changes of eight land use/cover types using CLUMondo^57^. By contrast, Zhao et al. simulated the changes of only five land use/cover types using CLUMondo when assessing the effects of land use policies on ecosystem services^58^. Our proposed method facilitates the setting of conversion orders even if the number of land system types is larger, e.g., more than 20 in this study, and when demand–supply relationships are many-to-many, like in this study.

## Conclusions

CLUMondo is the only model that simulates land changes by incorporating the multifunctionality of a land system. This incorporation enables CLUMondo to support kinds of demands, both area and non-area, and to establish many-to-many relationships between diverse demands and different types of land systems, thus allowing a more realistic and useful simulation of land changes. For example, it has been used to explore not only the changes of land cover types but also land-use intensification e.g.,^7,27^. Therefore, it has found an increasing number of applications, where the simulation results serve as the basis of diverse analysis.

In this study, we first investigated the source code of CLUMondo, providing for this model’s complete, detailed mechanism. By doing so, we facilitate future improvement on CLUMondo and its deep coupling with other earth system models. More importantly, we found that the featured function of CLUMondo—balancing demands and supplies in a many-to-many mode—relies on a parameter called conversion order. This parameter should be set manually according to the characteristics of each study area and based on expert knowledge, which is not feasible for users without understanding the whole, detailed mechanism. Therefore, the second contribution of this study is the development of an automatic method for adaptively determining conversion orders. Users with the method no longer require expert knowledge and fine-tuning for any study area. We revised the source code of CLUMondo to incorporate the proposed automatic method. To demonstrate its validity and effectiveness, we performed comparative experiments using two representative case studies, i.e., Sichuan and Henan. To ensure the experiments involved the featured function of CLUMondo, we established land systems and many-to-many demand–supply relationships (10 demands met by the supply by more than 20–30 land systems) for simulation in both case studies. From these results, we made the following three conclusions:

- Our investigation into the complete, detailed mechanism of CLUMondo is successful in that it allows the identification of core parameters of the model and future improvements;
- Conversion order is a core parameter that affects the simulation performance of CLUMondo; the performance might be unacceptably poor if conversion orders are not well specified; and
- Our proposed automatic method for adaptively determining conversion orders is valid and highly effective.

We modified the source code of CLUMondo to integrate the proposed method as an option for users (who can still set conversion orders manually). To distinguish the modified CLUMondo from the official version, we referred to this modified one as CLUMondo-BNU v1.0 and also released it for public use. It is important to note that both the original and improved models rely on logistic regressions, and the focus of this study is not to improve the regression module. However, as one of the core modules of CLUMondo, regression is recommended to improve by future studies using advance techniques such as auto-models for spatially autocorrelated occupancy and abundance data^59^, the geographically weighted temporally correlated logistic regression model^60^, the Maximum Entropy Model (i.e., Maxent)^61,62^, artificial intelligence^63^.

## Supplementary Information

Supplementary Information.

## Data Availability

The source code for the model (i.e., CLUMondo-BNU v1.0) and its manual are archived on Zenodo (https://doi.org/10.5281/zenodo.7051199). All data used to produce the Sichuan results present in this paper are archived on Zenodo (https://doi.org/10.5281/zenodo.6594722), and that used to produce the Henan results present in this paper are also archived on Zenodo (https://doi.org/10.5281/zenodo.6594815).
