# Supplementary material for: CLUMondo-BNU for simulating land system changes based on many-to-many demand–supply relationships with adaptive conversion orders
Source: Sci Rep. 2023 Apr 5;13:5559. doi: 10.1038/s41598-023-31001-3 (PMC10076298; doi:10.1038/s41598-023-31001-3)
Supplement: Supplementary file 1 — Supplementary Information. [file 41598_2023_31001_MOESM1_ESM.docx]

**Manual of CLUMondo-BNU**

**Peichao Gao**^1,2^**, Yifan Gao**^2^ **, Xiaodan Zhang**^2^ **, Sijing Ye**^1,2^**, and Changqing Song**^1,2,*^

^1^ State Key Laboratory of Earth Surface Processes and Resource Ecology, Beijing Normal University, Beijing, 100875, China. ^2^ Center for Geodata and Analysis, Faculty of Geographical Science, Beijing Normal University, Beijing, 100875, China

Contact Y.F. Gao for help on using CLUMondo-BNU: gaoyifan@mail.bnu.edu.cn

**0 Introduction**

The CLUMondo-BNU is an improved version of the CLUMondo model. Compared to the original version of the CLUMondo model, we add automatically determining the conversion orders of different land systems based on their capability for supplying a specific service. The method is powerful in that it is effective in improving the simulation accuracy of CLUMondo, efficient in operation, and widely applicable.

**1 Download the CLUMondo-BNU**

In order to make it convenient for users to learn and run the CLUMondo-BNU, we have provided the source code on Zenodo(10.5281/zenodo.7051199). The source code includes C++ source code (.cpp files) and the header file (.h files). The following content will tell the users how to download and run the CLUMondo-BNU.


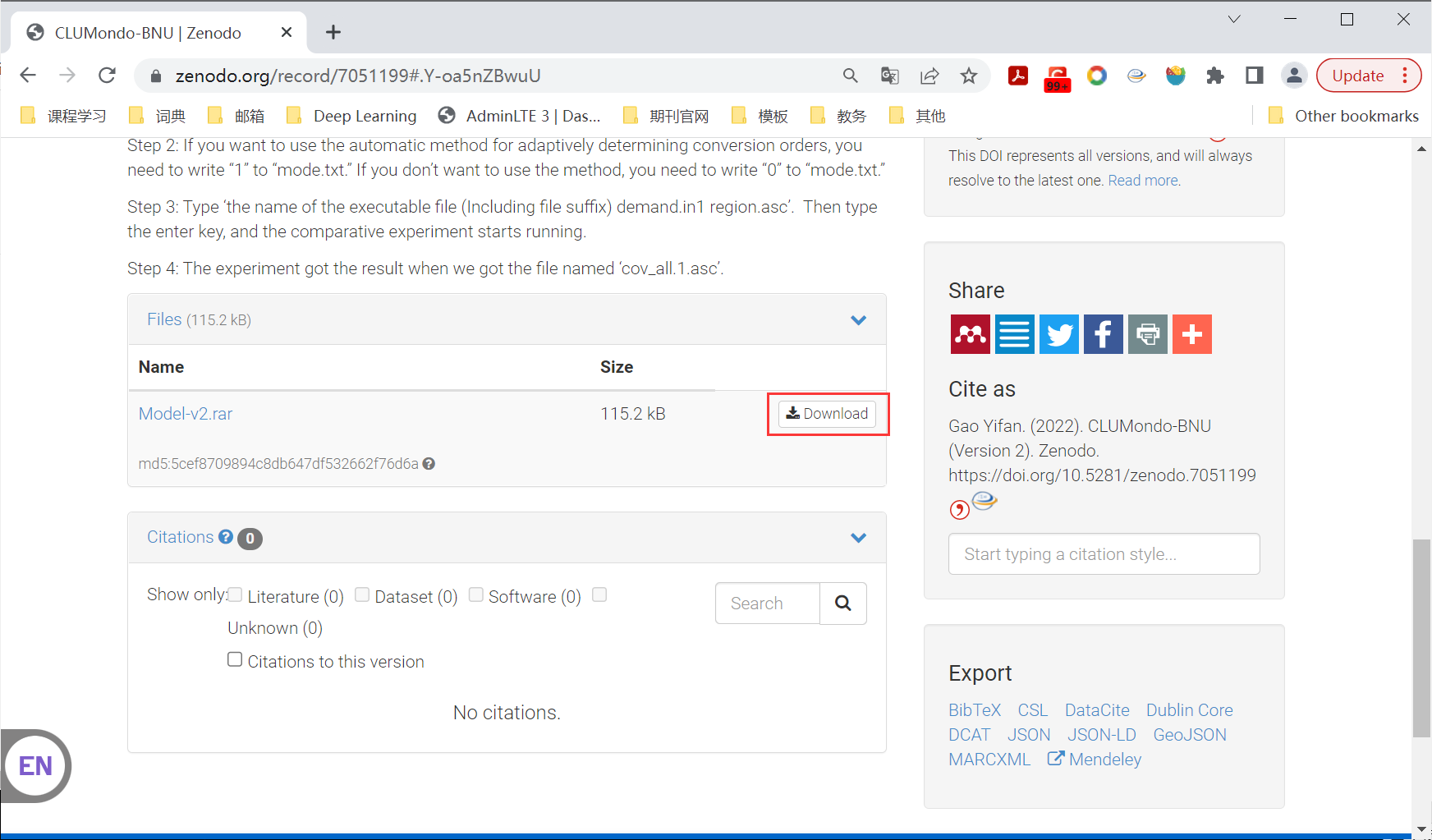


**2 Preparation before using the CLUMondo-BNU**

These instructions will get you a copy of the project up and running on your local machine for development and testing purposes.

CLUMondo-BNU is based C++ and Python. If you want to run the CLUMondo-BNU, you need to install the C++ environment in Visual Studio and install Python in your computer. In addition, you need to configure the environment so that Visual Studio can execute Python code. The specific preparations are as follows:

**Install the C++ environment in Visual Studio**

You can verify that Visual Studio is installed by creating a new project.

Step1: You can click [File][New][New Project] to creating a new project.


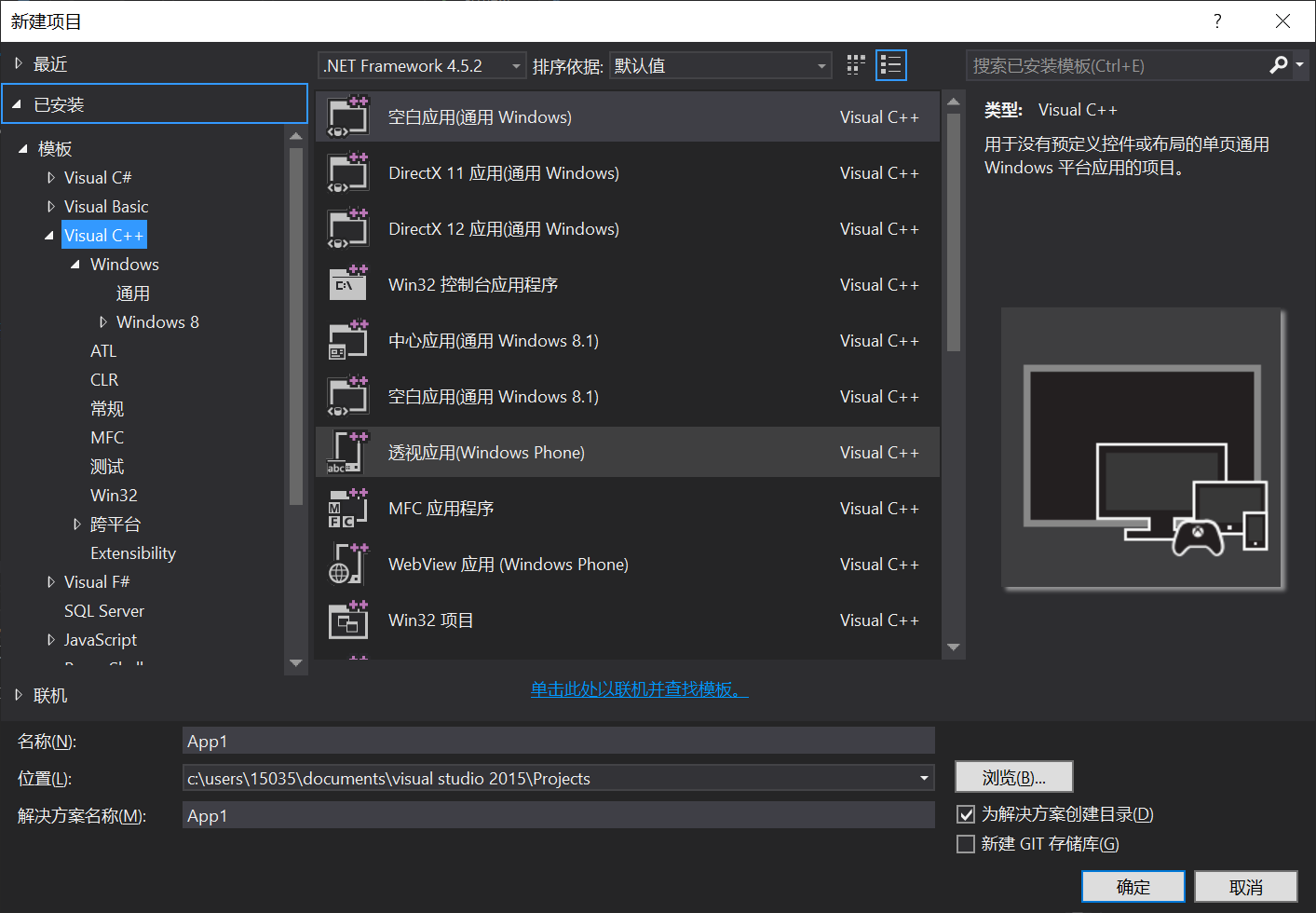


Step2: In the wizard of creating a new project, you can select the [Visual C++]. If the wizard prompts you to install the C++ environment, follow the installation wizard to complete the installation.

**Install Python in your computer**

If you want to install Python in your computer, please download the Python in the URL (<https://www.python.org/downloads/>). After downloading the Python, you can double-click the file you download. According to the tips for installation steps, you can finish to install Python in your computer.

In addition, jenkspy, numpy and os packages in Python also need to be installed. You can install these packages in CMD.exe. You can run the pip command when installing these packages. The following figure is an example of installing the jenkspy package.


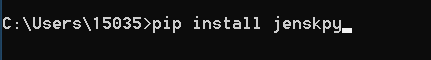


**Generate C++ projects from C++ code using CMake software**

Step1: download the CMake(<https://cmake.org/>).


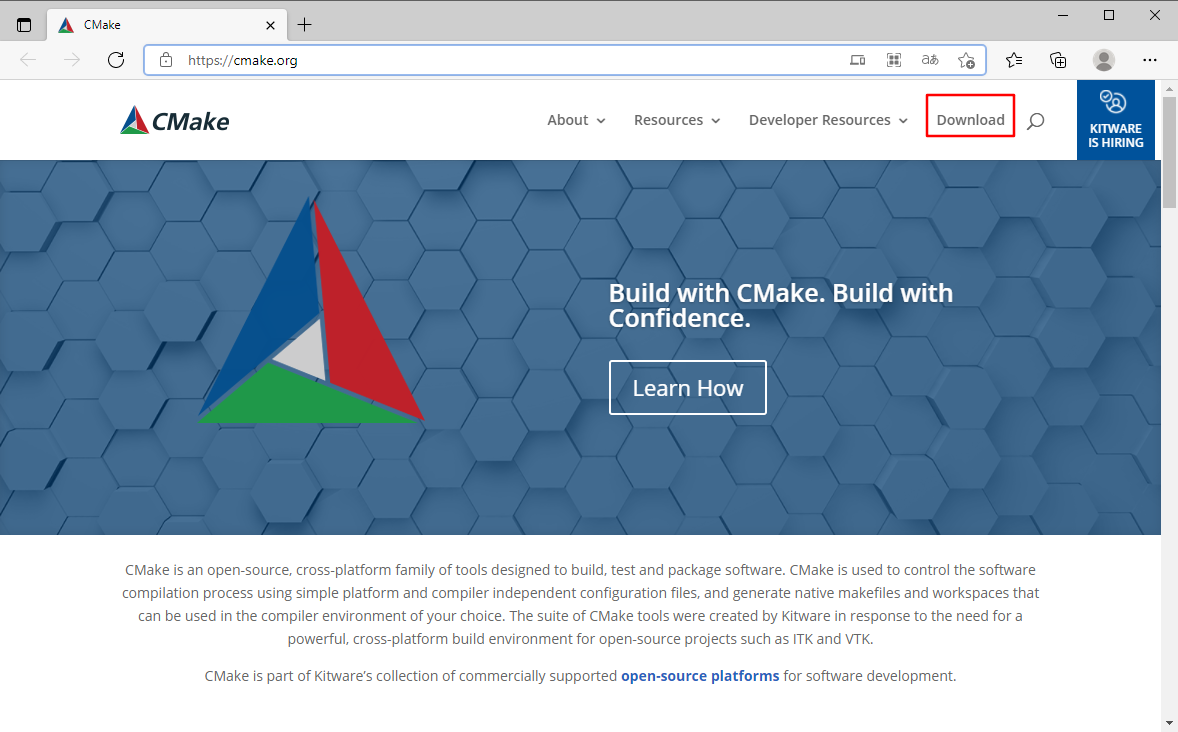


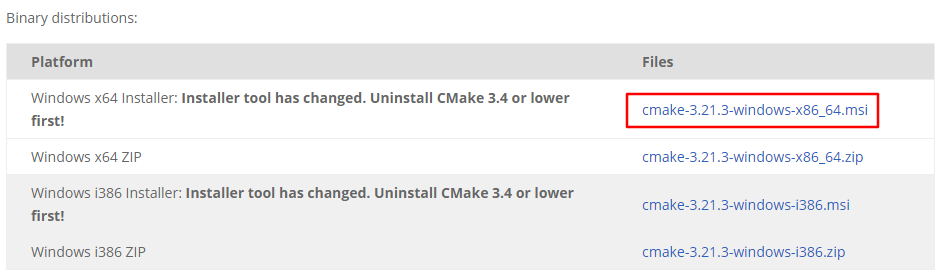


Step2: follow the installation wizard to install.

Step3: Open the CMake software, load the folder where the cpp file is located in [Where is the source code]. Then under this folder, create a new folder [biuld], and load the folder into [Where to build the binarias].


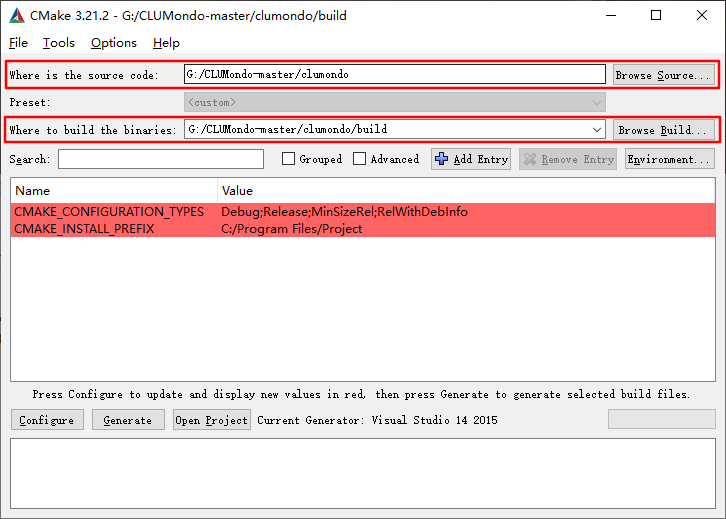


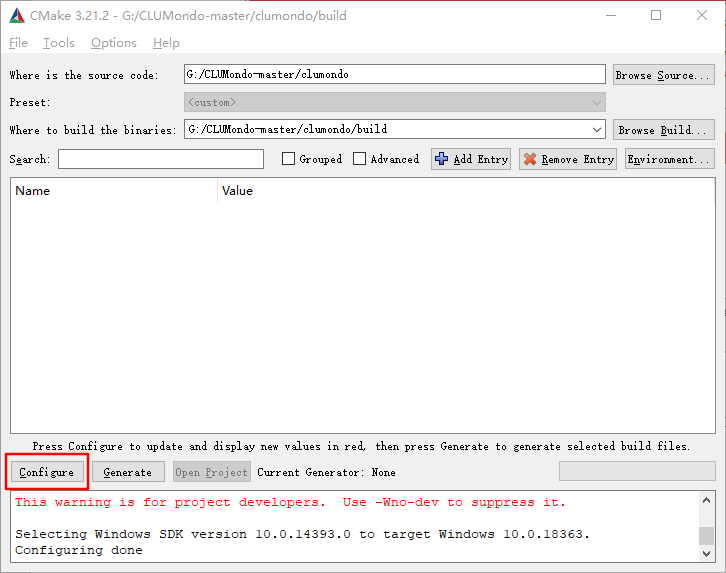


Step4: Select the version of Visual Studio installed on your computer, as well as the optional platform for generate.


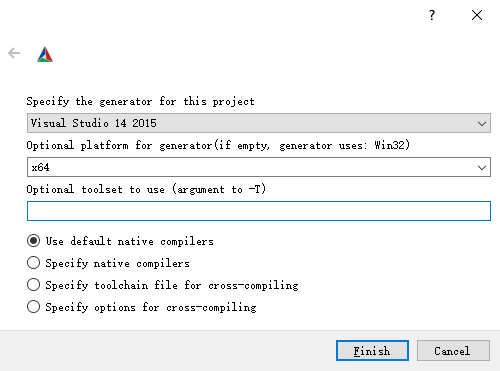


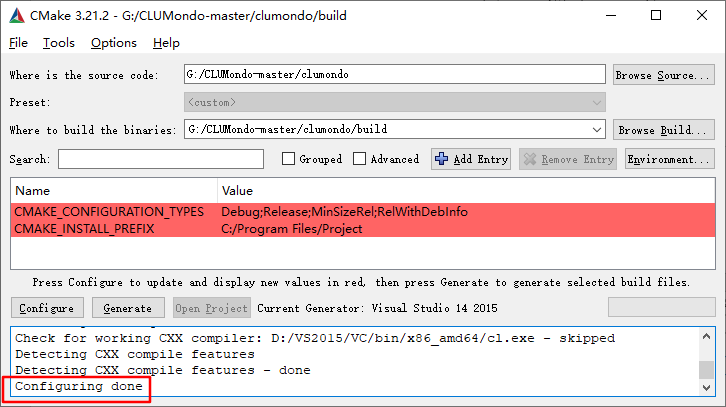


The CMake shows “Configure done” when the configuration is complete.

Step5: Click the [Generate].


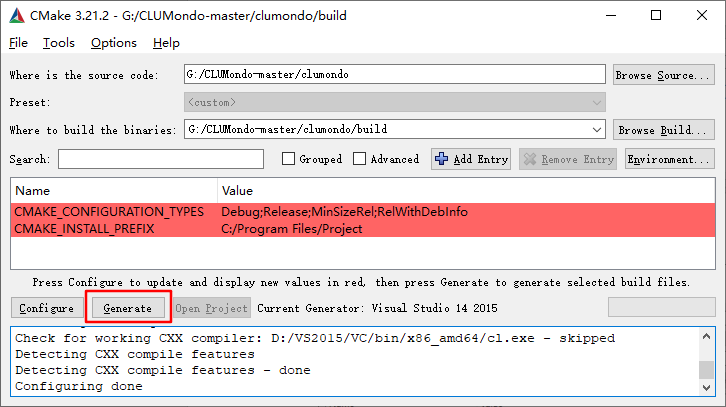


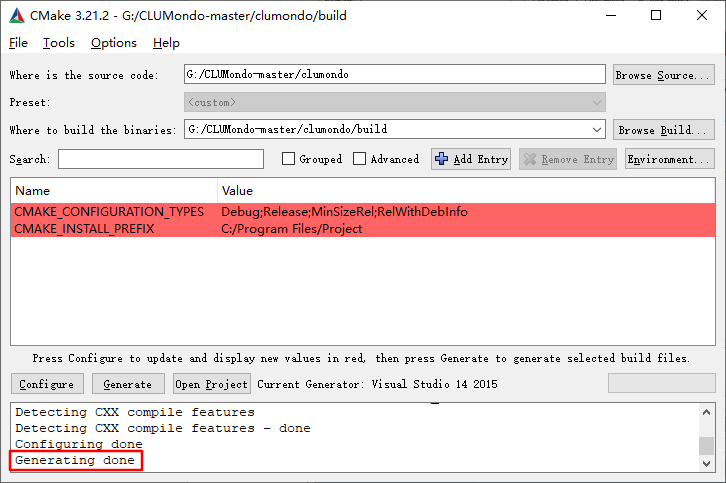


Then, you can using the Visual Studio to open the source code of the CLUMondo-BNU.


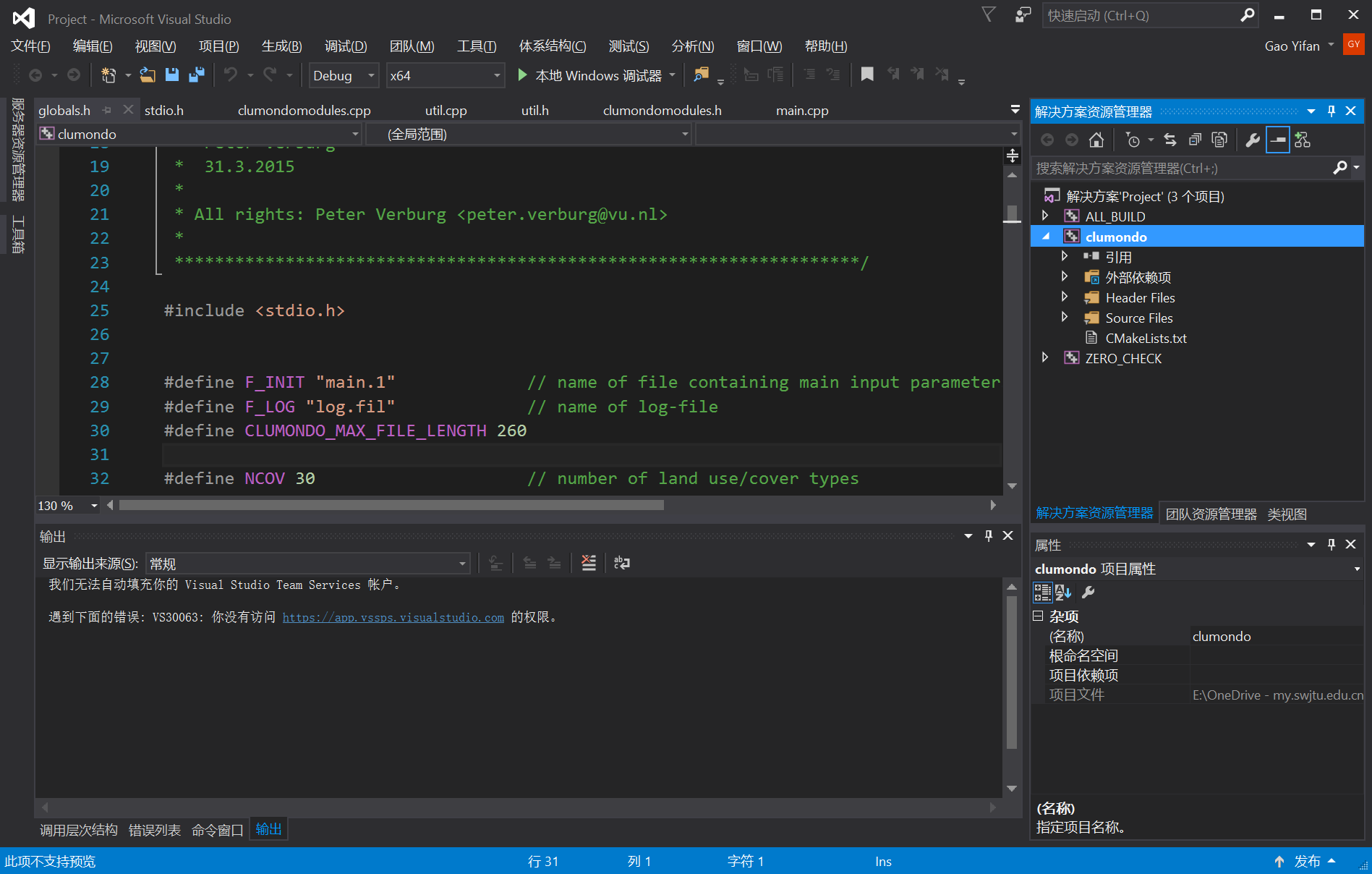


**Configure the environment so that Visual Studio can execute Python code**

Step1: You should right-click the project to open the properties, and add the include folder (such as C:\Users\15035\AppData\Local\Programs\Python\Python39\include) of the python installation location in the configuration properties - C/C++ - general - additional include directory.


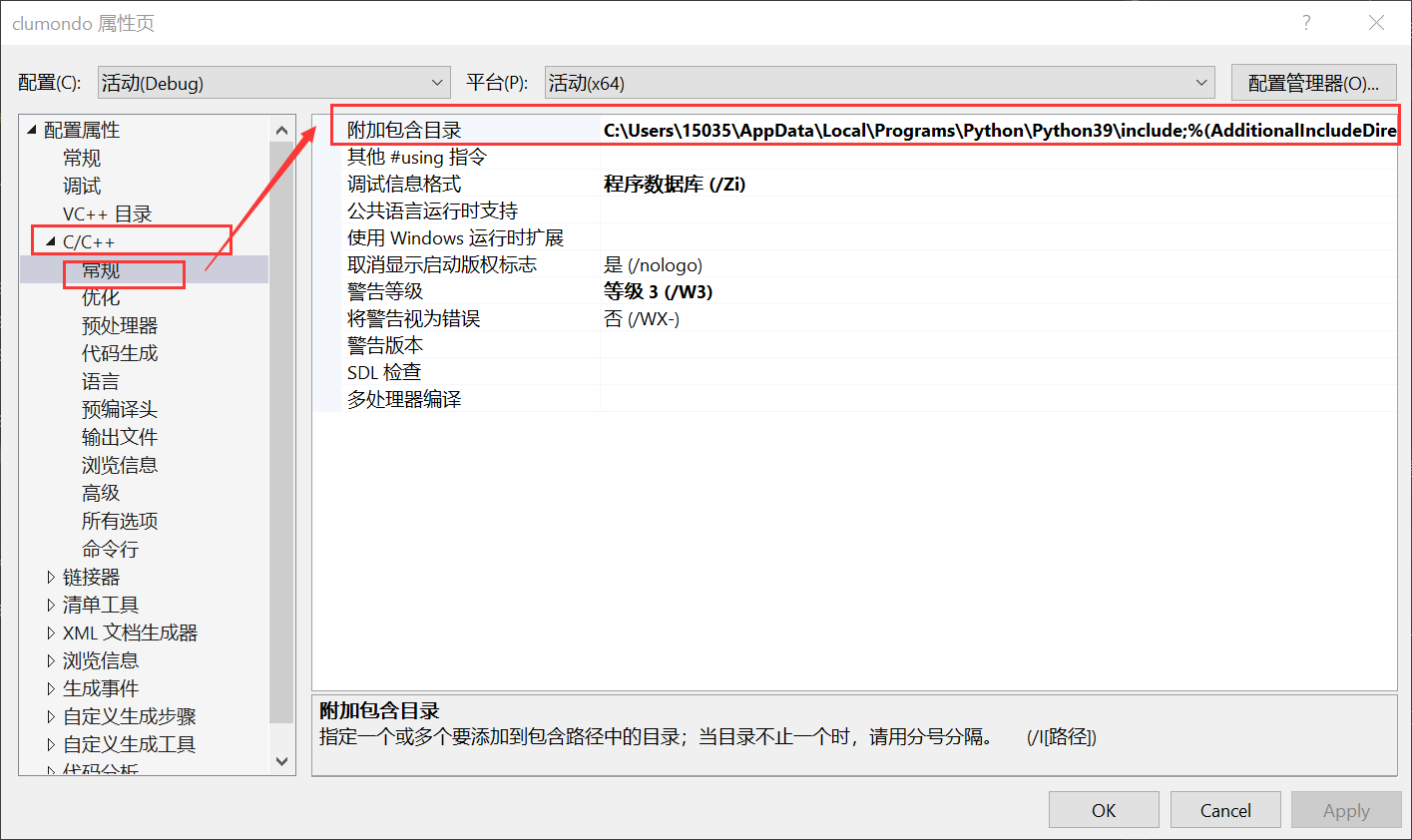


Step2: You need to add Python's libs folder (such as D:\Python\Python39\libs) under Configuration Properties - VC++ Directory - Library Directory.


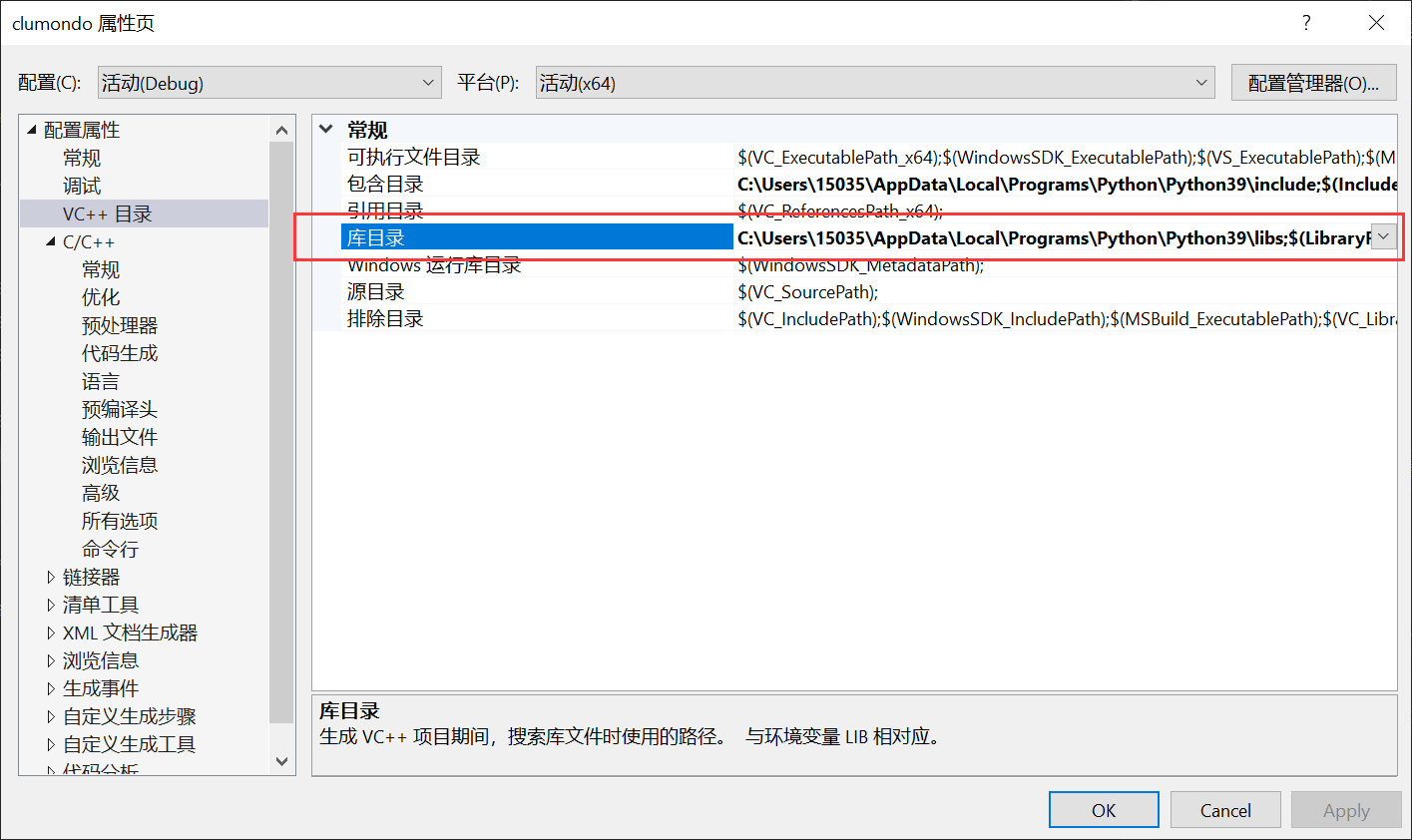


**Build the executable**

Build the solution in Visual Studio, then get the executable file. In addition, you need to copy the break.py file and python39.dll to the directory where the executable is.


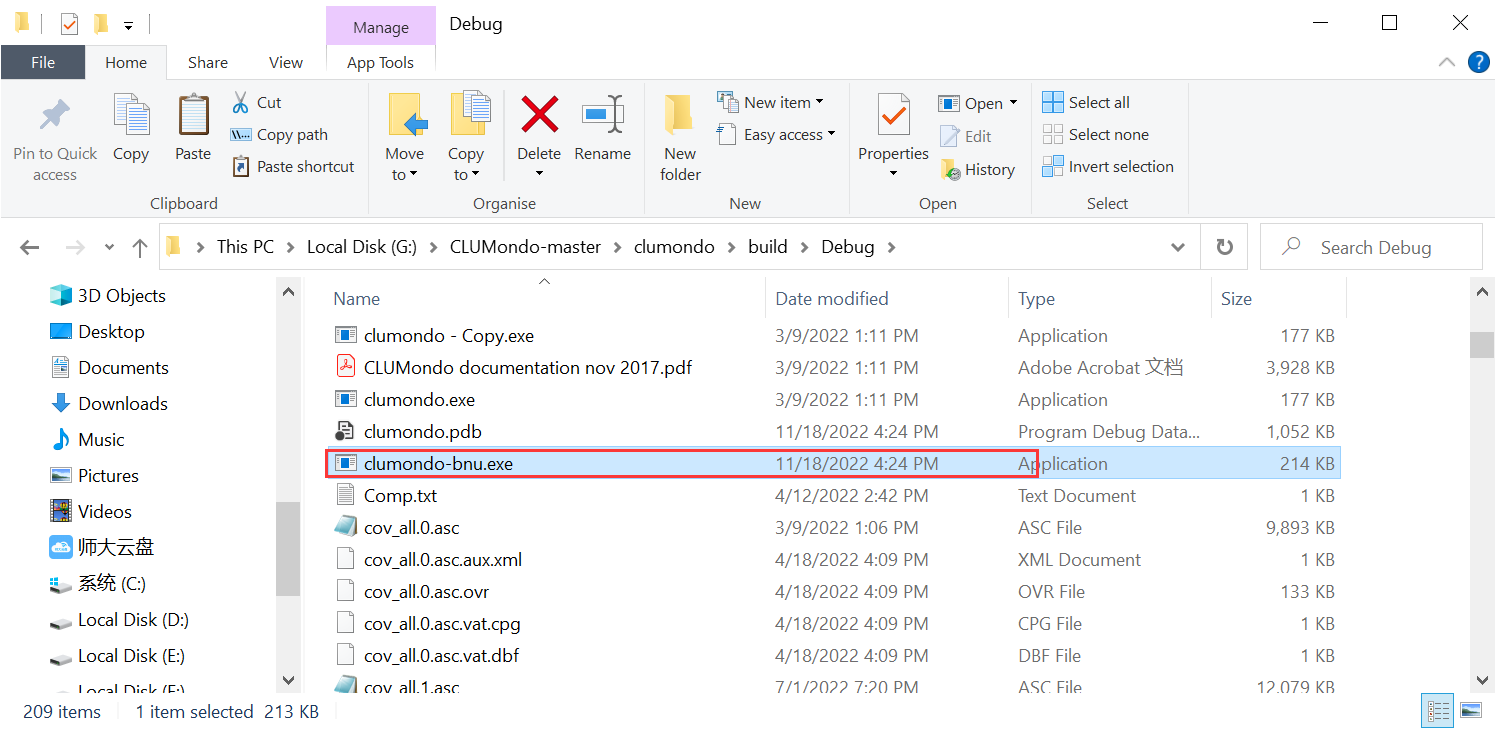


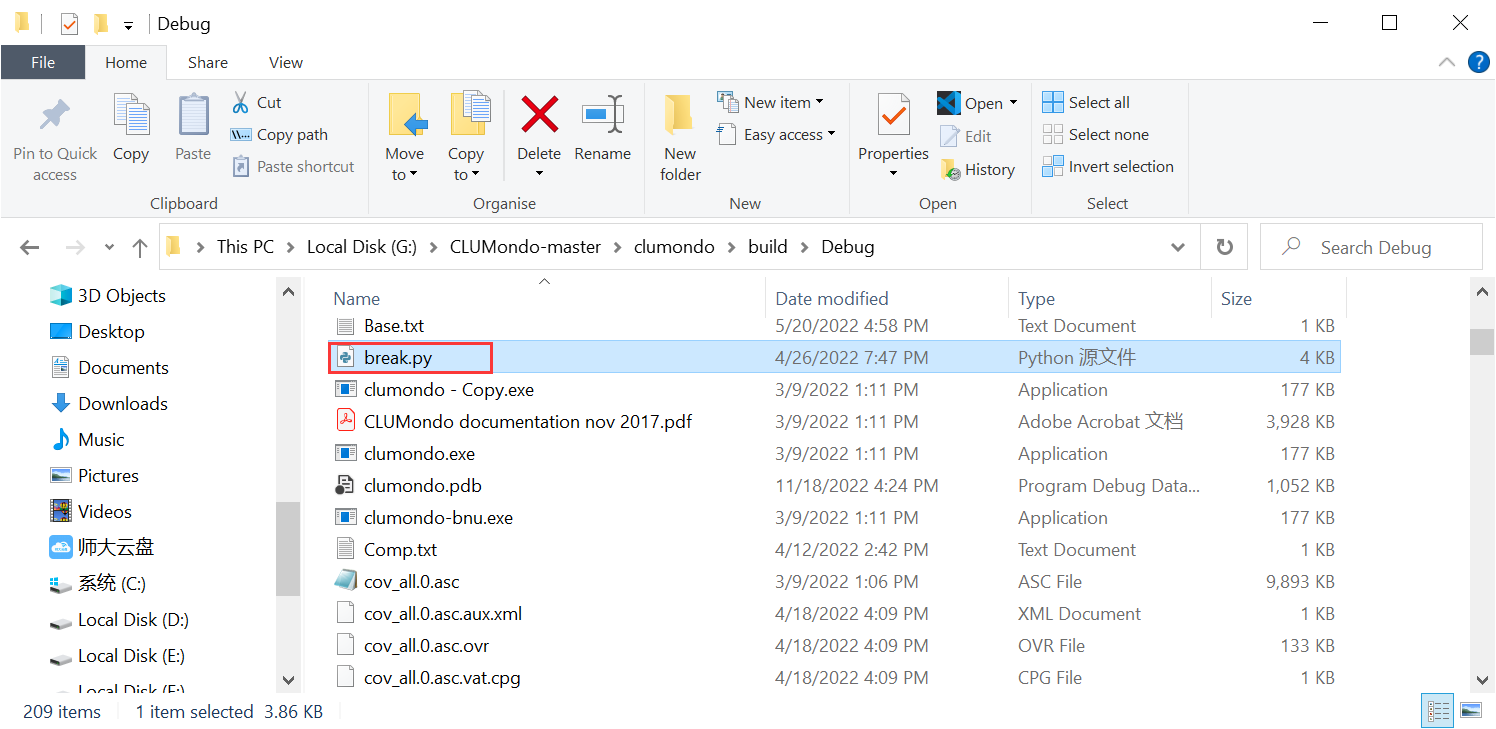


**3 Running the CLUMondo-BNU**

The following steps show you how to run the CLUMondo-BNU software(“CLUMondo-BNU.exe”).

Running CLUMondo-BNU requires the creation of a series of input files that meet the requirements of CLUMondo-BNU. In order to facilitate the use of CLUMondo-BNU, we provide the processed data in the 10.5281/zenodo.6594815(the data of Henan Province) and 10.5281/zenodo.6594722(the data of Sichuan Province).

The specific steps are as follows:

Step1: Copy the data in ‘Data\Comparative\CLUMondo_exe_and_mannual’(you can download from 10.5281/zenodo.6594815 or 10.5281/zenodo.6594722) to the folder where the “CLUMondo-BNU.exe” is located.

Step2: Decide whether to run CLUMondo-BNU. If you want to run the CLUMondo-BNU, you need to write “1” in “mode.txt” in ‘Data\Comparative\CLUMondo_exe_and_mannual’. (In addition, if you want to run the CLUMondo model, you need to write “0” in “mode.txt”.)


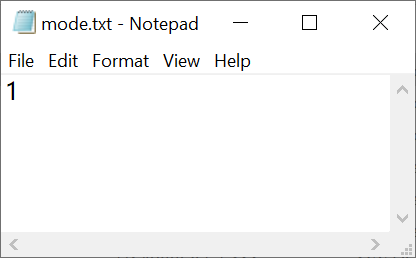


Step3: Open the CMD.exe, and modify the current path of CMD.exe to the path stored by the executable file in Step1.


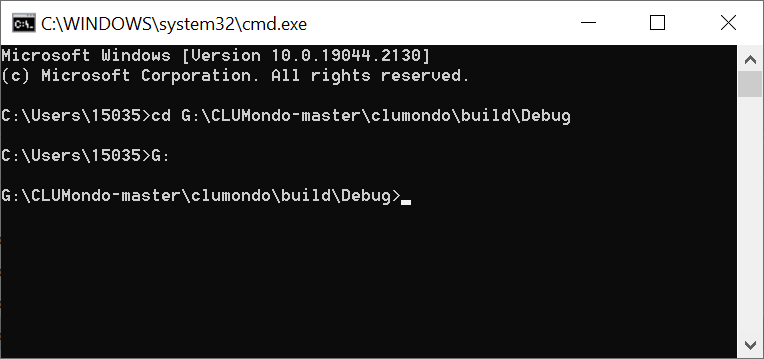


Step3: Type ‘the name of the executable file (The name of the executable file should include file suffix. For example, you can type “CLUMondo-BNU.exe”) demand.in1 region.asc’. Then type the enter key and the comparative experiment starts running.


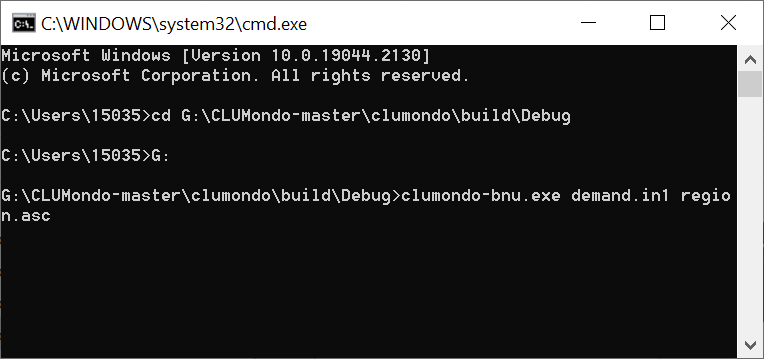


Step4: The comparative experiment got the result when we got the file named ‘cov_all.1.asc’.


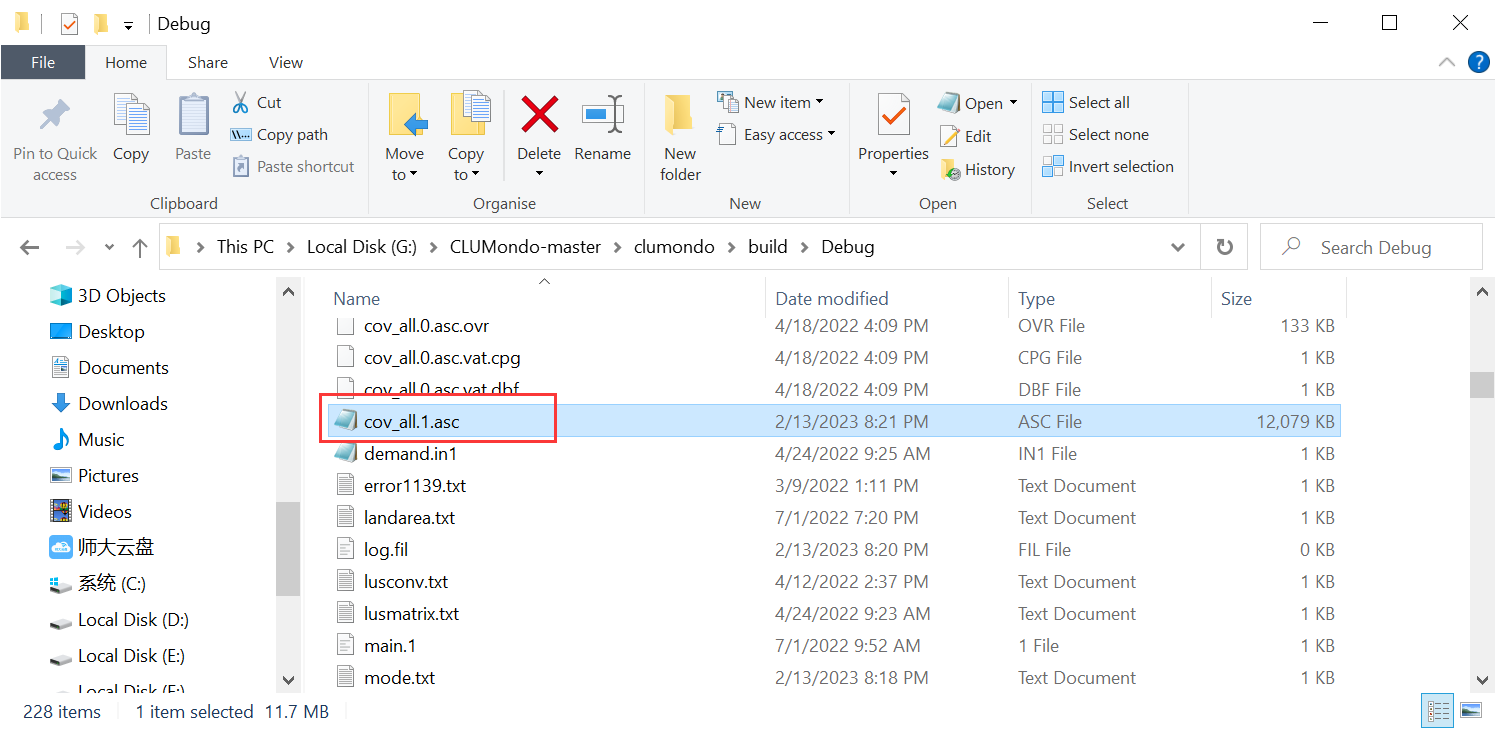


**4 More information**

The requirements of a series of input files for the CLUMondo-BNU is the same as the command line version of the CLUMondo. If you want to learn the particular requirements, you can visit the <https://www.environmentalgeography.nl/site/data-models/models/clumondo-model/>.


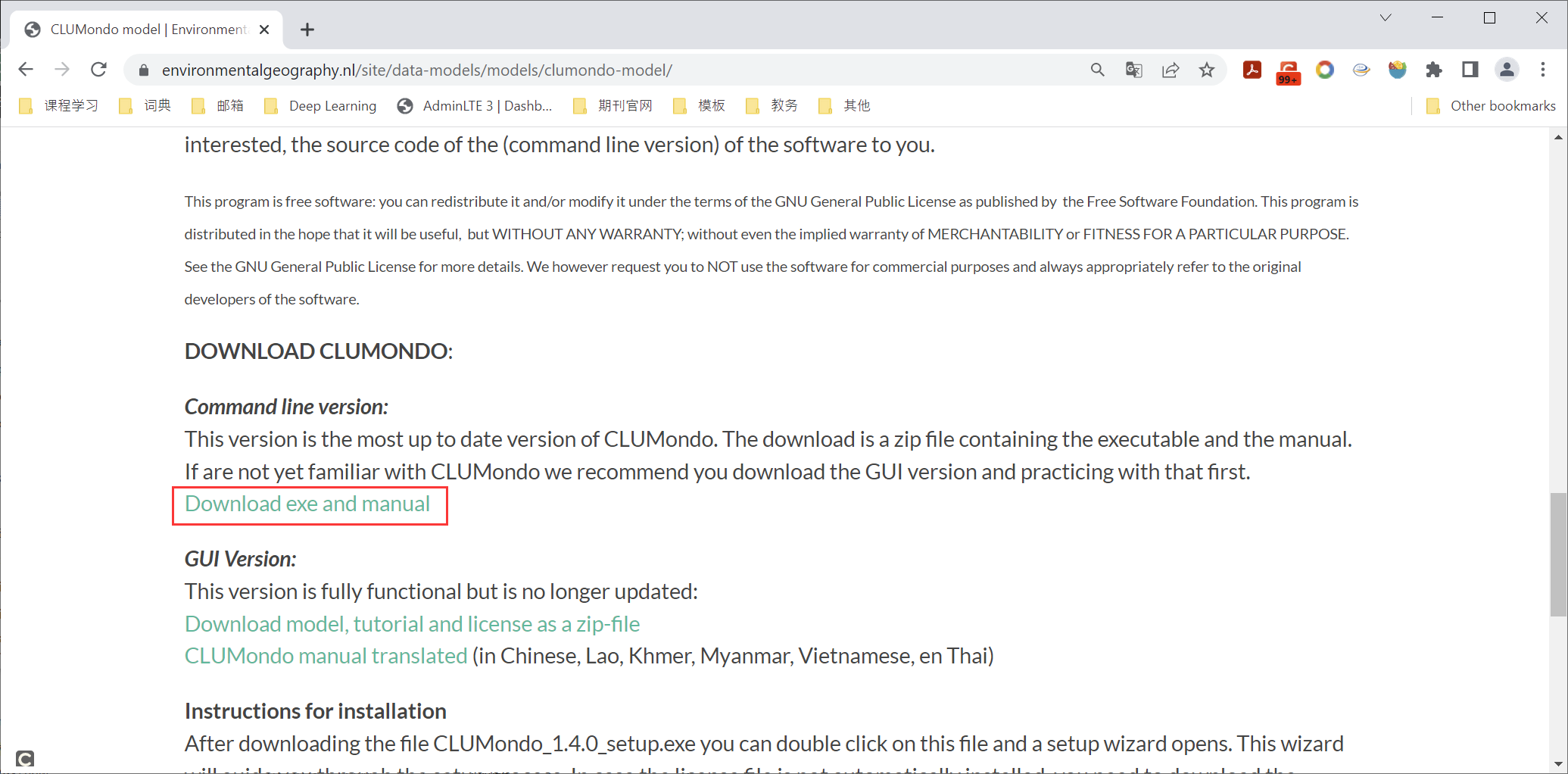


In addition, you can learn the source code of the CLUMondo from Github(https://github.com/VUEG/CLUMondo).
